# In silico lineage tracing through single cell transcriptomics identifies a neural stem cell population in planarians

**DOI:** 10.1186/s13059-016-0937-9

**Published:** 2016-04-27

**Authors:** Alyssa M. Molinaro, Bret J. Pearson

**Affiliations:** Hospital for Sick Children, Program in Developmental and Stem Cell Biology, Toronto, ON Canada; Department of Molecular Genetics, University of Toronto, Toronto, ON Canada; Ontario Institute for Cancer Research, Toronto, ON M5G0A4 Canada

**Keywords:** Single cell RNAseq, Waterfall, in silico lineage tracing, Neural stem cells, νNeoblasts, Planarians, *Schmidtea mediterranea*

## Abstract

**Background:**

The planarian *Schmidtea mediterranea* is a master regenerator with a large adult stem cell compartment. The lack of transgenic labeling techniques in this animal has hindered the study of lineage progression and has made understanding the mechanisms of tissue regeneration a challenge. However, recent advances in single-cell transcriptomics and analysis methods allow for the discovery of novel cell lineages as differentiation progresses from stem cell to terminally differentiated cell.

**Results:**

Here we apply pseudotime analysis and single-cell transcriptomics to identify adult stem cells belonging to specific cellular lineages and identify novel candidate genes for future in vivo lineage studies. We purify 168 single stem and progeny cells from the planarian head, which were subjected to single-cell RNA sequencing (scRNAseq). Pseudotime analysis with Waterfall and gene set enrichment analysis predicts a molecularly distinct neoblast sub-population with neural character (νNeoblasts) as well as a novel alternative lineage. Using the predicted νNeoblast markers, we demonstrate that a novel proliferative stem cell population exists adjacent to the brain.

**Conclusions:**

scRNAseq coupled with in silico lineage analysis offers a new approach for studying lineage progression in planarians. The lineages identified here are extracted from a highly heterogeneous dataset with minimal prior knowledge of planarian lineages, demonstrating that lineage purification by transgenic labeling is not a prerequisite for this approach. The identification of the νNeoblast lineage demonstrates the usefulness of the planarian system for computationally predicting cellular lineages in an adult context coupled with in vivo verification.

**Electronic supplementary material:**

The online version of this article (doi:10.1186/s13059-016-0937-9) contains supplementary material, which is available to authorized users.

## Background

Understanding how adult stem cells (ASCs) are regulated in homeostatic conditions and how they respond to injury and disease is a crucial step in the advancement of regenerative medicine [1]. In particular, elucidating the transition of cell states during lineage progression is a necessary precursor to developing techniques for the directed differentiation of tissue-specific ASCs. In vivo lineage tracing by transgenic labeling has proven to be a key experimental technique for studying the progressive changes that occur as a stem cell differentiates to produce a mature cell type [2]. However, the limited number of ASCs present in vertebrate tissues makes the study of adult lineage progression difficult in these organisms [3].

The freshwater planarian *Schmidtea mediterranea* is a non-parasitic flatworm well known for its regenerative ability [4–6]. Planarians have a large population of ASCs, termed neoblasts, which comprise approximately 20 % of the cells in the animal and are collectively responsible for the homeostatic maintenance and regeneration of all tissue types [7, 8]. Although *S. mediterranea* is morphologically simple, molecular studies involving in situ hybridizations of a variety of neural markers have demonstrated complexity within the planarian central nervous system (CNS) [9–14]. The planarian CNS consists of a bi-lobed brain comprised of approximately 5000 neurons that exist in precise patterns and ratios of major neuronal subtypes [13–15]. Two ventral nerve cords extend posteriorly to the tail tip of the animal and the animal has an extensive peripheral nervous system [16]. Not only can a decapitated planarian regenerate its entire brain in 7–10 days, but it has recently been shown that an uninjured animal has high levels of neuronal cell death and replacement (homeostasis) [17, 18]. Together, this has led to the hypothesis that there may be a population of ASCs committed to producing cells required by the CNS (i.e., neural stem cells) [12, 19].

Although planarians have the advantage of complete, scarless neural regeneration and provide the ability to study ASC biology in vivo, they have not been amenable to genetic lineage tracing experiments used in other model systems. Thus, it has been a major challenge to understand the cellular lineage progression from a parental ASC to differentiated neurons. A candidate gene approach is typically used where gene function is removed by RNA interference (RNAi), regeneration or homeostasis defects assayed, and the resulting lineage changes pieced together in a temporally backwards manner [12, 18–21]. As an alternative, unbiased approach, here we demonstrate that lineages can be computationally determined through the use of single-cell sequencing of planarian stem cells and their division progeny. Recently, a newly described bioinformatics approach called Waterfall was applied to single-cell RNA sequencing (scRNAseq) data obtained from transgenically labeled neural stem cells to study their progression from quiescence to activation [22]. By temporally arranging single cells based on their gene expression profiles, Waterfall is able to order cells as a continuum of transient states that define the progression of a particular lineage. Due to the ease of stem cell and progeny purification in *S. mediterranea* [18, 23], we hypothesize that Waterfall can be applied to study lineage progression in planarians as an in silico lineage-tracing tool.

Here we present scRNAseq of purified planarian stem (X1) and progeny (X2) cells specifically isolated from the head region and demonstrate the usefulness of the Waterfall analysis pipeline to study neural lineage progression in this model system. Hierarchical clustering of the scRNAseq dataset revealed a high degree of heterogeneity within the planarian head and allowed for the identification of distinct groups of cells based on gene expression profiles. One group, which we have termed the “ν (nu) Neoblasts”, exhibited overrepresentation of gene sets associated with neural processes and reduced expression of some stem cell and cell cycle genes. By using known markers of planarian stem cells and markers previously shown to be highly expressed in the brain, we were able to identify and exclude the cell clusters that were not involved in neuronal differentiation and subsequently perform pseudotime analysis on the remaining cells to reveal a putative progression through transient states along a neural lineage. To validate the proposed lineage, Waterfall was used to visualize temporal changes in the expression of many other known stem cell and neural markers and showed that they decrease and increase, respectively, over pseudotime for this proposed lineage. Further, several genes previously undescribed in planarians with high expression in the νNeoblasts were identified and shown by fluorescent in situ hybridization (FISH) to be expressed in a novel *piwi-2*^+^*piwi-1*^*lo*^ cycling stem cell sub-class in the head. In this way, we demonstrate the usefulness of computational transcriptome analysis with Waterfall to develop testable hypotheses about cell-state transitions even in very heterogeneous datasets and demonstrate that solving lineages with scRNAseq is a strength of the planarian system.

## Results

### Single-cell RNAseq reveals a high degree of stem cell heterogeneity in the planarian head

**Fig. 1:**
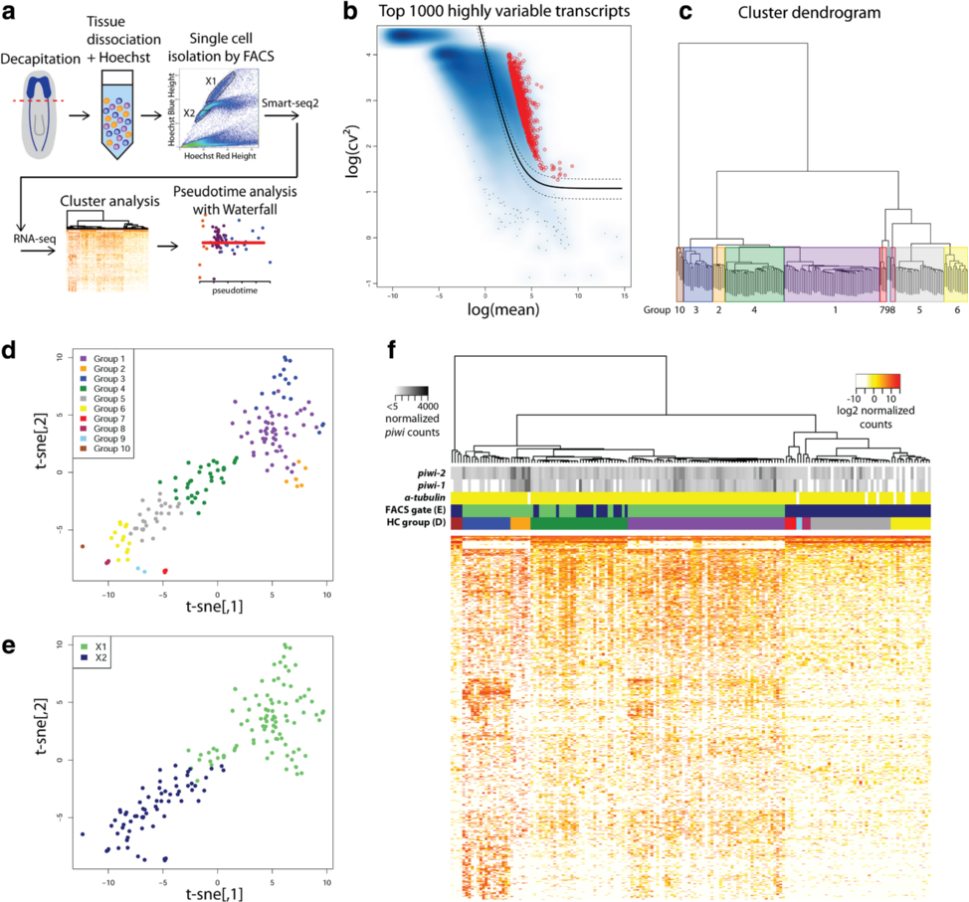
Hierarchical clustering of single-cell expression profiles identifies ten subgroups in the head. **a** Overview of the single-cell RNAseq pipeline. Planarian heads were amputated at the posterior extent of the brain lobes and dissociated. Cells were stained with Hoechst and single cells were isolated by FACS. cDNA libraries were prepared and sequenced and reads were aligned to the SmedASXL transcriptome assembly. Cluster and pseudotime analyses were subsequently performed. **b** Transcripts were plotted according to their mean expression level and coefficient of variation across all single-cell samples and ranked by significance of their deviation from the fit. The top 1000 highly variable transcripts are circled in *red*. The *solid line* is the regression line; *dashed lines* indicate the 95 % confidence interval. **c** Hierarchical clustering (HC) based on the top 1000 highly variable transcripts identified ten subgroups in the planarian head (*colored boxes*, *group number* indicated below). **d, e** t-Distributed Stochastic Neighbor Embedding (*t-sne*) plots of single cells colored by HC group membership (**d**) or FACS gate (**e**). **f** Heatmap of log2 normalized gene expression for the top 1000 highly variable transcripts in each single cell sample. *Color bars*: HC group, group membership colored as in (**d**); FACS gate, colored as in (**e**); *alpha-tubulin*, *yellow* indicates detection; *piwi-1* and *piwi-2* normalized expression counts are in *greyscale* according to the *color key* at the *upper left*

scRNAseq was used to assess the level of neoblast heterogeneity in planarian heads (Fig. 1a). Neoblasts are thought to be the only cycling cells in planarians and are irradiation-sensitive [24, 25]; thus, these cells are ablated within 24 h following exposure to 60–100 Gray of γ-irradiation. Due to the rapid rate of cell turnover [8], immediate, differentiating progeny of stem cell divisions are also lost shortly thereafter [23]. These characteristics were used to set gates for fluorescence-activated cell sorting (FACS) as previously described (Additional file 1: Figure S1) [18, 23]. FACS was used to isolate 96 individual stem cells (hereafter “X1s”, >2C DNA content) and 72 individual early progeny cells (hereafter “X2s”, <2C DNA content) from the planarian head region based on Hoechst fluorescence, along with three tube controls of 200 cells each (two X1 and one X2). cDNA libraries were prepared for each sample using SmartSeq2 and libraries were tagmented using the Nextera XT kit to allow for multiplexed sequencing [26]. Single-cell libraries were sequenced to an average depth of 4.5 million reads and reads were aligned to the SmedASXL transcriptome assembly using bowtie2 [27], yielding a 64 % average alignment rate. Bulk samples were sequenced to a depth of 10–20 million reads. On average, 5150 transcripts were detected in each X1 cell and 2050 transcripts were detected in each X2. At least 1000 transcripts were detected in >98 % of cells. To ensure that this cell isolation strategy captured cells of all lineages known to be present in the planarian head, expression of known lineage-specific markers was considered. Markers for epithelium, gut, protonephridia, muscle, neurons, eyes, and pharynx were detected in the bulk control samples (Additional file 2: Figure S2a). In addition, examples of single cells expressing markers of specific lineages were identified (Additional file 2: Figure S2b).

The dataset was reduced to include only the top 1000 highly variable transcripts for all subsequent analyses (Additional file 3: Supplemental data file 3). This was accomplished by selecting transcripts with the highest variation in expression levels across the single cell samples. Because dropout events can be a common source of technical variation in single cell cDNA synthesis [28, 29], we placed the additional constraint that these transcripts must also have a relatively high mean expression level (log_2_(mean) > 3 normalized counts) (Fig. 1b). Hierarchical clustering of the single cell expression profiles revealed a high degree of heterogeneity among both X1s and X2s and groups were defined by cutting the dendrogram at an arbitrary height that allowed for separation of both X1 and X2 populations into distinct groups (ten total groups; Fig. 1c). To validate the initial cluster analysis t-Distributed Stochastic Neighbor Embedding (t-SNE) [30] was used to plot a two-dimensional representation of the scRNAseq dataset. t-SNE clustered the cell groups in agreement with hierarchical clustering (Fig. 1d). As expected, t-SNE also showed clear separation of the X1 and X2 cells (Fig. 1e), further validating the clustering results.

The results of the hierarchical clustering analysis on the top 1000 most variable transcripts are summarized in Fig. 1f. Of the ten groups, Groups 1–3 consisted entirely of X1s, Groups 5–10 consisted entirely of X2s, and Group 4 contained both X1s and X2s. Detection of the ubiquitous *Smed-α-tubulin* was used as a positive control for gene detection in all cells and the well-described stem cell markers *piwi-1* and *piwi-2* were used to validate X1 identity [31]. Interestingly, while *piwi-2* was detected in 100 % of X1s, *piwi-1* was only very lowly detected or absent in the expression profiles of Group 3 cells. Because the mean expression level of *piwi-1* among single X1 cells was 1685 ± 24 normalized counts (3866 ± 48 counts per transcript per million reads in previously published bulk X1 data [18, 32]), it was unlikely that the low detection in Group 3 was due to dropout events during library preparation. Importantly, neither *piwi-1* nor *piwi-2* were identified computationally to belong to the 1000 most variable transcripts used for clustering, indicating that the clustering of cells with low *piwi-1* expression is representative of a true biological stem cell state and not an artifact of the gene set used for clustering. As previously observed, *piwi-1* and *piwi-2* expression was variable among sorted X2 cells [31]. In total, these scRNAseq data from head X1 and X2 cells suggested high molecular heterogeneity, as well as a novel X1 type (Group 3) which had a novel *piwi-2*^*+*^*piwi-1*^*lo*^ expression state.

### Gene set enrichment analysis reveals an X1 population in the head enriched for neural gene sets

**Fig. 2:**
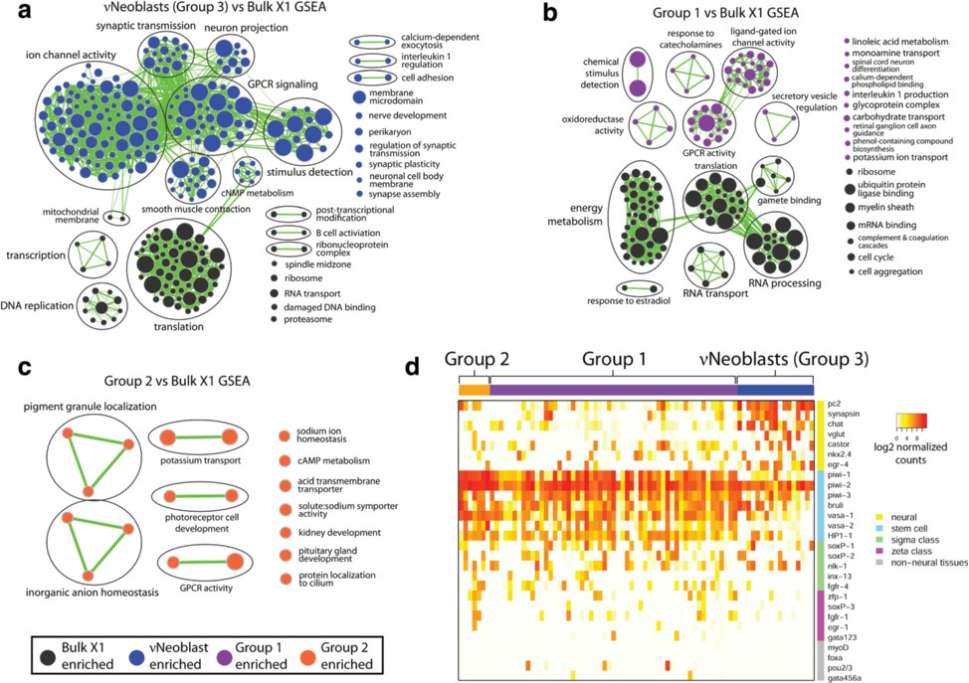
Neural gene sets are enriched in some groups compared with bulk X1 data. **a**–**c** Gene set enrichment analysis (*GSEA*) results for Group 3 (FDR < 0.01, *p* < 0.001) (**a**), Group 1 (FDR < 0.05, *p* < 0.001) (**b**), and Group 2 (FDR < 0.05, *p* < 0.001) (**c**). *Nodes* represent gene sets and node size is proportional to the GSEA nominal enrichment score. *Node color* represents the group in which the gene set is enriched. The width of the *green edges* represents the number of genes shared between the connected nodes. Similar gene sets are *circled* and their function is labeled. Group gene expression profiles are averages of the single-cell expression profiles in the group. **d** Heatmap displaying log2 normalized counts of known markers for neural, stem cell, sigma class, zeta class, and non-neural tissue. *Columns* are single cells from the group noted above the heatmap. *GPCR* G protein-coupled receptor

Gene set enrichment analysis (GSEA) was performed to determine whether any groups were enriched for gene sets associated with neuronal processes when compared with the multiple datasets on whole-body bulk X1 sequencing replicates [18, 32]. Because there is currently no annotated database for planarian genes, those with reciprocal BLAST hits to mouse homologs (e < 1e^-4^) were identified and the corresponding mouse gene IDs were used for GSEA as previously described [32]. Notably, the *piwi-2*^*+*^*piwi-1*^*lo*^ Group 3 displayed an overrepresentation (false discovery rate (FDR) < 0.01; *p* < 0.001) of neural-related gene sets (Fig. 2a), such as “neuron projection”, “synaptic transmission”, and “nerve development”; thus, Group 3 will now be referred to as the “nu-Neoblasts” (νNeoblasts). Group 1 was also enriched for neural-associated gene sets (Fig. 2b). Group 2 was enriched for very few gene sets compared with the bulk X1 data and these gene sets were predicted to be involved in a range of cellular processes, including several neural-associated processes [33] (Fig. 2c). Interestingly, neither Group 4 nor any of the X2-only groups were enriched for neural-associated gene sets, suggesting that maturing neurons may not pass through the X2 gate.

In order to understand these X1 sub-groups in more depth, expression levels of several known neural and stem cell transcripts were examined (Fig. 2d). Compared with Groups 1 and 2, νNeoblasts exhibited the highest expression of the pan-neural markers *pc2* [34] and *synapsin* [10] as well as other genes known to be expressed in the brain (listed in Fig. 2d). Conversely, νNeoblasts exhibited relatively low expression of known stem cell markers, such as *vasa-1* [35] and *HP1-1* [36], although expression of these genes was still detected. Expression of non-neural tissue progenitor markers (*myoD* (muscle) [12], *foxa* (pharynx) [37], *pou2/3* (protonephridia) [20] and *gata456a* (gut) [38]) was detected in a minority of Group 1 cells but was absent from all νNeoblasts, suggesting that the νNeoblasts may represent an X1 population responsible for specifically contributing to neuronal lineages. Notably, Group 2 cells exhibited the highest expression of stem cell markers, especially *piwi-1* and *piwi-2*, and did not express non-neural tissue markers, suggesting that Group 2 may be the least committed to any lineage.

### Waterfall analysis predicts a neural lineage trajectory

**Fig. 3:**
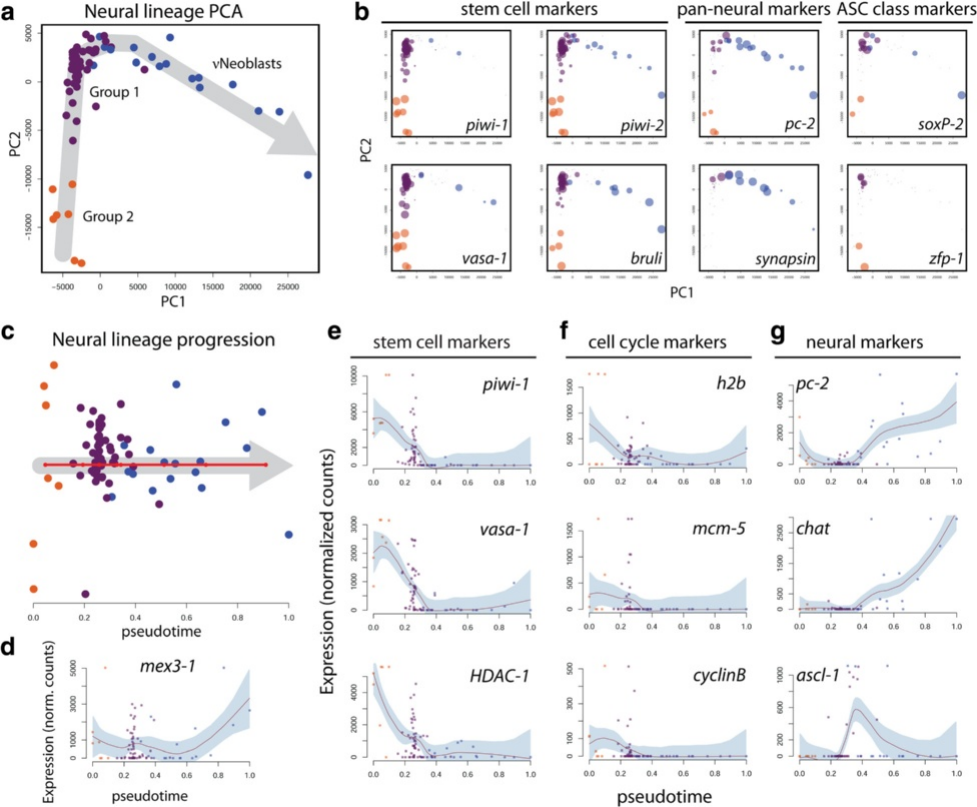
Pseudotime analysis with Waterfall predicts a neural lineage trajectory. **a** PCA plot for Groups 1 and 2 and νNeoblasts. *Grey arrow*, proposed lineage orientation; each *point* is a single cell. **b** PCA plots with data point size proportional to the expression level of the gene specified in each plot. **c** Plot of Groups 1 and 2 and νNeoblast cells ordered along pseudotime. *Red line*, linearized minimum spanning tree connecting k-mean centers; *grey arrow*, direction of lineage progression; *y-axis*, distance of cell to its nearest k-means center. **d**–**g** Expression patterns of known markers support the Waterfall lineage prediction: **d** differentiation gene *mex3-1*; **e** stem cell markers *piwi-1*, *vasa-1*, and *HDAC-1*; **f** cell cycle markers *h2b*, *mcm-5*, and *cyclinB*; **g** neural markers *pc-2*, *chat*, and *ascl-1. Red line*, local polynomial regression fit; *shaded region*, 95 % confidence interval

Pseudotime analysis with Waterfall provides an unbiased approach for reconstructing lineages from single cell transcriptome data with a minimal requirement for prior knowledge of the lineage in question [22]. Here, Waterfall was used to predict a neural lineage trajectory from the scRNAseq dataset. Principal components analysis (PCA) was initially performed on all ten hierarchical clustering groups; however, the first two principal components (PC1 and PC2) primarily separated the highly heterogeneous X2 groups, resulting in poor resolution of the X1 groups from which all lineages are expected to originate (Additional file 4: Figure S3) [17]. Because the X2s were not enriched for neural gene sets by GSEA, all of the X2-containing groups were removed and PCA was performed for the remaining cells (i.e., X1 only; Fig. 3a). While the results provided good resolution of the X1 groups, the number of lineages and their orientations were not immediately clear. For instance, one could imagine a trajectory beginning with the νNeoblasts and progressing through Group 1 then Group 2, or the exact opposite. It is also possible that Group 1 represents the earliest stem cell state, which can differentiate along two distinct lineages (Group 2 or νNeoblast). To predict the most probable orientation of the trajectory, known stem cell and neural markers were examined and relative expression is represented as the size of the data points in the PCA plots in Fig. 3b. From this it was evident that Group 2 cells consistently displayed the highest expression of the known stem cell markers *piwi-1*, *piwi-2*, *vasa-1* and *bruli* [39]. Expression of these four genes remained high in some Group 1 cells but was only lowly detected in others. Finally, νNeoblasts generally did not express *piwi-1* or *vasa-1* but did express *piwi-2* and *bruli*. The expression levels of the pan-neural markers *pc-2* and *synapsin* were also considered for route determination. Both of these genes were consistently highly expressed in νNeoblasts and also in some Group 1 cells, but detection was lower or absent in Group 2 cells. Expression of proposed markers for the previously described σ and ζ neoblast classes was also considered. The ζ-class marker *zfp-1* was detected in only very few cells and did not appear to be group-specific [20] (see “Discussion”). Interestingly, expression of the σ-class marker *soxP-2* was detected primarily in Group 1 cells; however, analysis of scRNAseq data published by Wurtzel et al. [40] raises questions about the specificity of previously described σNeoblast markers. This is demonstrated in Additional file 5: Figure S4, which includes plots of single X1 cells isolated from the prepharyngeal region of uninjured animals obtained from an online resource published by Wurtzel et al. [40] (http://radiant.wi.mit.edu/app; see “Discussion”).

Overall, the expression analyses strongly predicted a lineage trajectory beginning with Group 2 and progressing through Group 1 followed by the νNeoblasts; this trajectory was subsequently used for pseudotime analysis. To temporally arrange the cells and assign pseudotime values, k-means clustering was performed on the PCA plot and the k-means centers were connected by a minimum spanning tree (MST) trajectory. A pseudotime value for each cell was subsequently computed as described by Shin et al. [22], which essentially flattens Fig. 3a into Fig. 3c. As a proof of principle that pseudotime analysis with Waterfall is a valid method for predicting cellular lineages, Waterfall was also applied to the well-characterized epithelial lineage using the scRNAseq data from [40]. The resulting pseudotime trajectory correctly predicted the temporal expression patterns of the epithelial lineage, beginning with ζ-class neoblasts, then early progeny, and ending with known late progeny markers (Additional file 6: Figure S5). Thus, pseudotime analysis with Waterfall is a valid method for predicting cellular lineage trajectories.

Plotting the expression levels of known genes along pseudotime illustrated the progressive changes that occur along the predicted νNeoblast neural lineage trajectory. Expression of *mex3-1*, a gene highly expressed in X1 and X2 cells and required for differentiation of neural cell types [18], was detected in all three groups and increased toward the end of pseudotime, which was the expected result because these cells are predicted to represent transient states along a continuum of increasing differentiation (Fig. 3d). Importantly, known stem cell markers showed expression that was highest early in pseudotime and then gradually decreased (Fig. 3e). Similarly, the cell cycle markers *h2b* [41], *mcm-5* [36], and *cyclinB* [31] were highest in Groups 1 and 2 and low in νNeoblasts despite the fact that all groups were sorted through the same X1 FACS gate (Fig. 3f). The expression of neural genes known to be expressed in the bulk X1 population, *pc-2* and *chat*, was initially low in pseudotime and began to increase in the last Group 1 cells, reaching a maximum in the νNeoblasts (Fig. 3g). Finally, expression of the *achaete-scute* gene homolog *ascl-1*, which has previously been shown to have X1 expression, peaked at the Group 1 to νNeoblast transition, further supporting a transition state in the predicted lineage and suggesting that neural fates are downstream of *ascl-1*, similar to its established roles in vertebrates and flies [12, 42–44]. Together with the GSEA results, analysis of the scRNAseq data with Waterfall confidently predicted the progression of a neural lineage through pseudotime based on the expression of known stem cell, cell cycle, and neural genes.

### *piwi-2* marks a population of head-specific stem cells

**Fig. 4:**
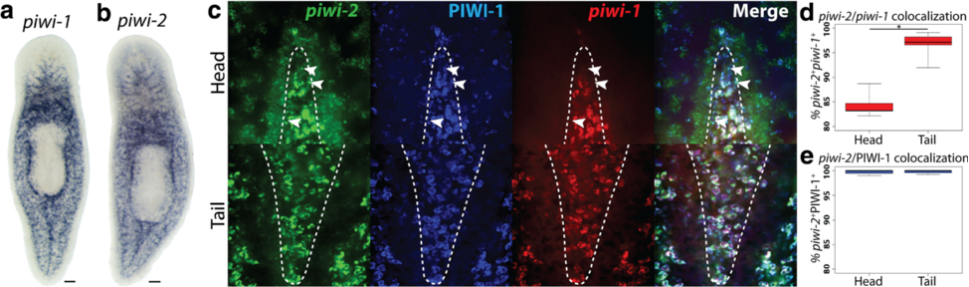
In situ identification of *piwi-2* ^+^ *piwi-1* ^lo^ stem cells. **a** Colorimetric WISH of *piwi-1*. **b** Colorimetric WISH of *piwi-2*. Scale bars = 100 μm. **c** High magnification images of the stem cell regions in the head between the brain lobes (*top row*) and tail stripe (*bottom row*) are shown. *Arrowheads*, *piwi-2* ^+^ *piwi-1* ^-^PIWI-1^+^ cells. **d** Quantification of *piwi-2* and *piwi-1* colocalization in the stem cell areas in the head and tail; n = 5, *p* = 0.00035. **e** Quantification of *piwi-2* and PIWI-1 colocalization in the stem cell areas in the head and tail; n = 5, *p* = 0.89742. Regions included in counts are outlined by a *dashed line* in **c**

The observation made during the initial cluster analysis that some head X1s expressed *piwi-2* but not *piwi-1* was surprising and warranted further investigation in vivo. Characterization by whole-mount in situ hybridization (WISH) demonstrated that *piwi-2* expression labeled more cells in the anterior than *piwi-1* along with diffuse brain labeling (Fig. 4a, b)*.* Because these cells were clustered together into the νNeoblast group, it was hypothesized that *piwi-2*^+^*piwi-1*^lo^ stem cells may be specific to the neural lineage; thus, double-fluorescent WISH (dFISH) was performed to assess the level of colocalization between *piwi-1* and *piwi-2* in the stem cell compartment between the brain lobes and in the tail region, where there is no brain (Fig. 4c). In the tail, 96.6 ± 2.8 % of *piwi-2*^+^ cells were also *piwi-1*^+^; however, in the head, only 84.4 ± 2.6 % of *piwi-2*^+^ cells also expressed *piwi-1* (Fig. 4d; *p* = 0.00035).

PIWI-1 protein has be shown to persist in cells even when *piwi-1* expression can no longer be detected and colocalization of PIWI-1 with lineage-specific markers has been used to mark progenitor populations [18, 45]. Because νNeoblasts were predicted to arise from a *piwi-1*^*+*^ population, all *piwi-2*^*+*^ stem cells were expected to be PIWI-1^+^. Indeed, >99 % of the *piwi-2*^*+*^ cells both between the brain lobes and in the tail colocalized with PIWI-1 (Fig. 4e; *p* = 0.89742), supporting the predicted lineage and that the *piwi-2*^*+*^ cells were recently *piwi-1*^*+*^*.* The observation that not all stem cells expressed the putative planarian pan stem cell marker *piwi-1* and, indeed, that its expression was absent in a specific subpopulation of stem cells in the head may explain why a neural stem cell population has been elusive to detect in planarians.

### Pseudotime analysis and in vivo validation of νNeoblast-enriched genes

**Fig. 5:**
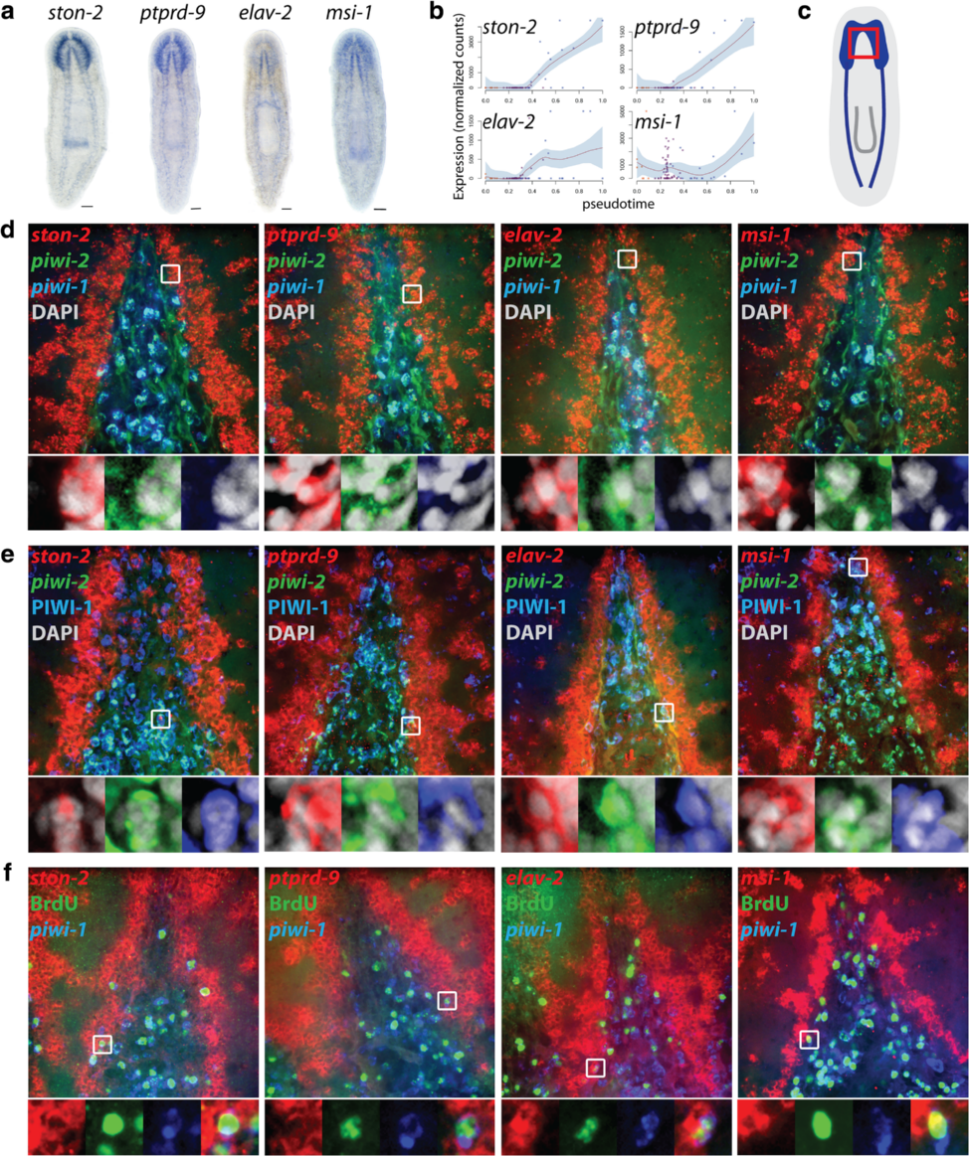
In situ validation of candidate neural lineage genes identified with Waterfall. **a** Colorimetric WISH. Dorsal view, anterior up, scale bars = 100 μm. **b** Pseudotime plots for genes in **a**. *Red line*, local polynomial regression fit; *shaded region*, 95 % confidence interval. **c** Diagram of the region imaged (*red box*) in **d**–**f. d** tFISH of each ν-gene with *piwi-2* and *piwi-1. Boxed regions* are magnified and displayed with DAPI below each image. **e** dFISH of each ν-gene with *piwi-2* and immunofluorescence for PIWI-1. *Boxed regions* are magnified and displayed with DAPI below. **f** dFISH of each ν-gene with *piwi-2* and immunofluorescence for BrdU following injection and a 4-h chase period. *Boxed regions* are magnified and displayed below. The *rightmost* high magnification panels are merged images

In order to identify novel candidate genes involved in neural lineage progression, the expression patterns of 11 genes enriched in the Group 3 νNeoblasts were characterized by WISH (Fig. 5a; Additional file 7: Figure S6; Additional file 8: Supplemental data file 4). Strikingly, every gene tested was expressed in the brain and many were also expressed in the ventral nerve cords and photoreceptors. Four of these genes (*ston-2*, *elav-2*, *ptprd-9*, and *msi-1* [46]), whose expression gradually increased over pseudotime (Fig. 5b), were further analyzed by triple FISH (tFISH) with *piwi-1* and *piwi-2* in the head (Fig. 5c)*.* Consistent with the observation that νNeoblasts generally did not express *piwi-1*, examples of *ν-gene*^+^*piwi-2*^+^*piwi-1*^lo^ cells in the stem cell compartment between the brain lobes were identified (Fig. 5d). In addition, *ν-gene/piwi-2* dFISH combined with immunofluorescence for PIWI-1 expression demonstrated the presence of *ν-gene*^*+*^*piwi-2*^*+*^PIWI-1^+^ cells in the head (Fig. 5e). In both cases, these cells were typically located along the lateral edge of the stem cell compartment, adjacent to the brain. This is consistent with the hypothesis that these cells arose from a *piwi-1*^+^ population (i.e., Group 1) and, as they continued to differentiate along the neural lineage, had begun to migrate toward the brain lobes where they will terminally differentiate into mature neurons. Further, homeostatic worms were injected with the thymidine analog bromodeoxyuridine (BrdU) and fixed after a 4-h chase period to determine whether these cells are actively cycling, which was predicted because they were isolated from the X1 gate. Following this very short time chase, some *ν-gene*^+^ cells had already incorporated BrdU (Fig. 5f). Interestingly, these cells typically also expressed low levels of *piwi-1*, which suggested that they represent the earliest stage of neural commitment.

**Fig. 6:**
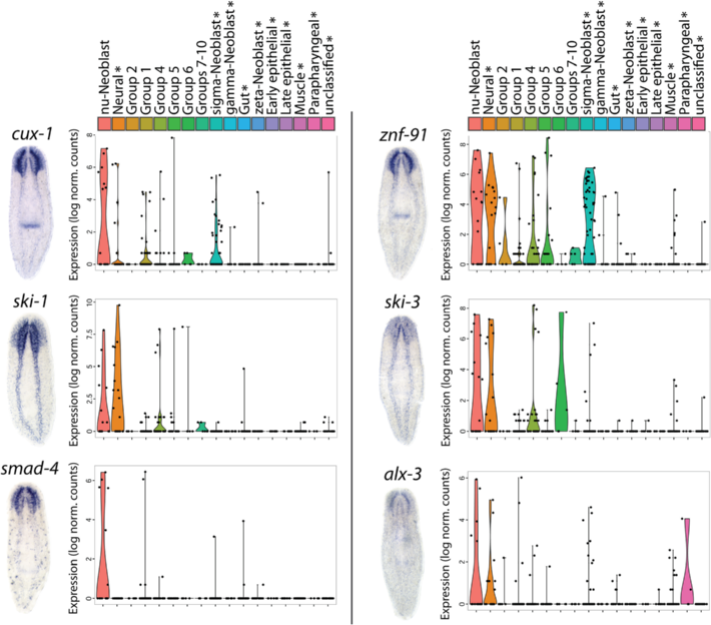
νNeoblast-enriched transcription factors have neural expression patterns and are largely specific to the neural lineage. *Left*: *cux-1*, *znf-91*, *ski-1*, *ski-3*, *smad-4*, and *alx-3* WISH. *Right*: violin plots displaying the expression levels of the corresponding transcription factor transcript in single cell groups identified in the current study (no *asterisks*) and in [40] (marked with an *asterisk*)

Additionally, six transcription factors enriched in νNeoblasts were identified and were shown to have primarily neural expression patterns by WISH (Fig. 6; Additional file 8: Supplemental data file 4). Comparison of the expression levels of these transcription factors between distinct cell groups identified in the current study as well as in [40] demonstrated that these genes are highly specific to νNeoblasts (X1s) and/or mature neurons (X-insensitive) (Fig. 6). Based on these in vivo validations of the in silico predictions, we concluded that pseudotime analysis with Waterfall can be used to identify new, lineage-specific expression differences, which can then provide the groundwork for future studies of neural homeostasis and regeneration.

### Waterfall analysis reveals a novel lineage trajectory through the X2 gate

**Fig. 7:**
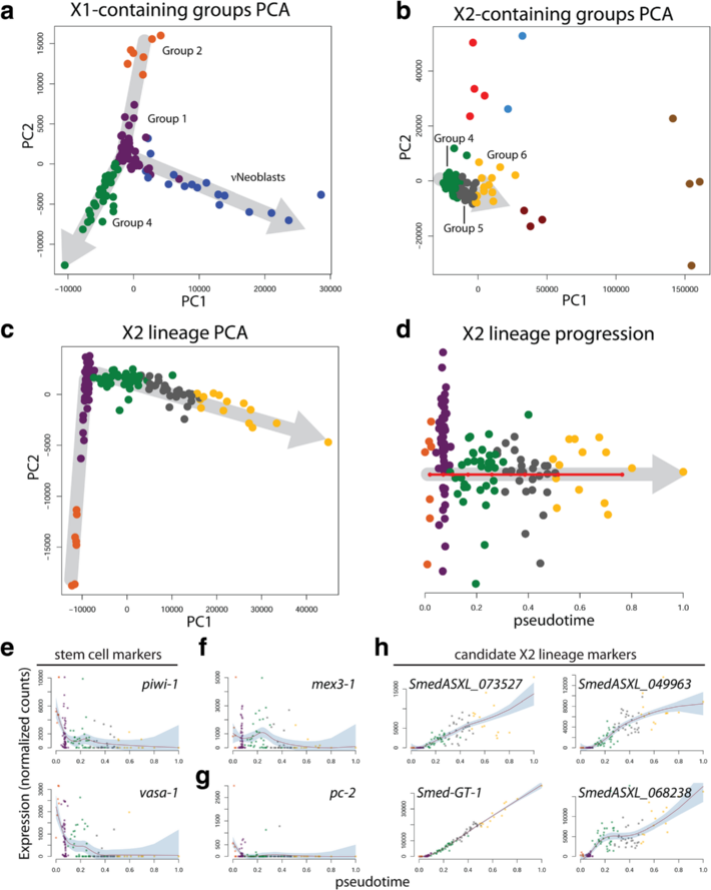
Pseudotime analysis with Waterfall predicts a novel, non-neural X2 lineage. **a** PCA plot for the X1-containing groups. **b** PCA plot for the X2-containing groups. **c** PCA plot for the predicted X2 lineage. **d** Plot of X2 lineage (Groups 1, 2, 4, 5, and 6) cells ordered along pseudotime. *Red line*, linearized MST connecting k-mean centers; *grey arrows*, direction of lineage progression; *y-axis*, distance of cell to its nearest k-means center. **e**–**g** Expression profiles of previously described genes in this potential X2 lineage: **e** stem cell markers *piwi-1* and *vasa-1*; **f** differentiation regulator *mex3-1*; **g** pan-neural marker *pc-2*. **h** Expression patterns of candidate markers for this predicted X2 lineage. *Red line*, local polynomial regression fit; *shaded region*, 95 % confidence interval

The initial Waterfall analysis on the full scRNAseq dataset suggested a prominent X2 lineage. Because Group 4 contained both X1 and X2 cells, we hypothesized that this group may represent cells transitioning from the X1 fraction to the X2 fraction. To understand how these cells related to the three X1-only groups, PCA was performed with the X2-only groups excluded (Fig. 7a). Interestingly, the Group 4 cells appeared to originate from Group 1 cells as a lineage separate from the νNeoblasts; this observation was also consistent with the hypothesis that Group 1 may represent a pluripotent stem cell population, from which multiple lineages originate. Next, 3in order to understand how Group 4 cells related to the X2-only groups, PCA was performed, this time excluding the X1-only groups. A clear trajectory through Groups 4, 5, and 6 was observed (Fig. 7b). Thus, Waterfall analysis predicted that Group 2 gives rise to Group 1, which can subsequently differentiate to produce νNeoblasts or proceed down the Group 4 to X2 lineage.

PCA and pseudotime analysis were performed for this predicted X2 lineage (Fig. 7c, d). As expected, expression of the stem cell markers *piwi-1* and *vasa-1* decreased over pseudotime (Fig. 7e). Expression of the differentiation-regulator *mex3-1* peaked at the Group 4 to Group 5 transition, which largely coincided with the transition from X1 to X2 (Fig. 7f). This suggested a role for *mex3-1* in directing X1 differentiation along an X2 lineage and agrees with the previous finding that *mex3-1* mediates the decision between self-renewal and differentiation [18]. The pan-neural marker *pc-2* was not detected in this lineage (Fig. 7g). Several examples of genes that gradually increased over pseudotime were identified by testing the most highly expressed transcripts in Group 6, as this group was predicted to be the most differentiated. Curiously, 11/18 of these transcripts did not have clear homologs in mice, humans, flies, or *C. elegans* but displayed striking pseudotime expression patterns (four representative examples are shown in Fig. 7h; Additional file 8: Supplemental data file 4).

The discrepancy in the average number of transcripts detected in X1s (5150) compared with X2s (2050) was consistent with the notion that stem cells are transcriptionally primed to produce several different cell types and that gene expression becomes more specific as cells differentiate. This concept was addressed by first comparing the number of transcripts detected in each cell with the number of sequencing reads, which revealed no correlation (Pearson correlation = 0.1869, *R*^2^ = 0.03494; Additional file 9: Figure S7a) and confirmed that the difference observed between X1s and X2s was not a consequence of data acquisition. Plotting the number of transcripts detected along pseudotime for the X2 lineage, which contained X1s and X2s, revealed that gene expression decreased gradually during this differentiation process (Additional file 9: Figure S7b, red dashed line indicates the beginning of lineage commitment). Again, this was independent of read depth, which remained constant throughout this pseudotime trajectory. As expected, this trend was less obvious for the neural lineage, which only extended to the earliest stage of lineage commitment and did not include any non-stem cell states (Additional file 9: Figure S7c).

Overall, Waterfall analysis has predicted the presence of a prominent, previously undescribed, non-neural X2 lineage in the planarian head, illustrating the sensitivity of this approach for lineage detection and demonstrating the strength of using unbiased techniques for discovery.

### Modeling planarian lineage relationships in silico

**Fig. 8:**
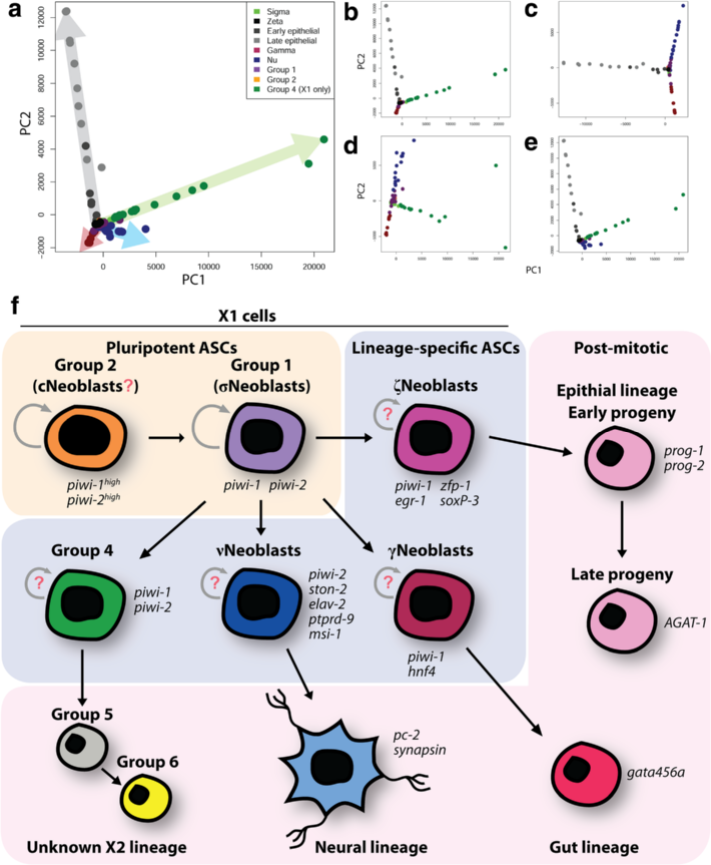
Model of planarian stem cell hierarchies. **a** PCA for predicted pluripotent and lineage-committed groups from the current study and [40]. *Colored arrows* indicate separate lineages. **b**–**e** PCA plots with the following lineages removed: **b** neural lineage; **c** novel X2/Group 4 lineage; **d** epithelial lineage; **e** gut lineage. **f** Summary model of planarian lineages. Based on the scRNAseq and Waterfall/pseudotime analyses, we hypothesize that cNeoblasts are represented in our Group 2 cluster, which give rise to pluripotent Group 1/σNeoblasts. In turn, σNeoblasts give rise to ζ, γ, ν, and Group 4 neoblasts, represented in the middle tier. We hypothesize that these neoblast subclasses give rise to tissue-specific lineages on the third tier, such as epithelium for ζNeoblasts, gut for γNeoblasts, and neurons for νNeoblasts. *Red question marks* denote either unknown existence or unknown ability to self-renew

Merging the scRNAseq datasets from the current study and [40], PCA was used to predict global relationships between the various groups identified by each study (using a newly generated list of the top 1000 highly variable transcripts from the combined datasets). Figure 8a displays a PCA plot including the predicted pluripotent groups (Groups 1 and 2 from the current study and the σNeoblasts from [40]) and groups representing various lineages (the νNeoblasts and Group 4 X1s from the current study and the γNeoblasts and epithelial lineage groups (ζNeoblasts, early epithelial and late epithelial) from [40]). The result was quite striking: Group 1, Group 2, and σNeoblasts clustered directly on top of each other and formed a vertex from which the lineage-specific groups extended outward as distinct, non-overlapping “branches”. Importantly, this “lineage tree” pattern was maintained even upon removing different lineages from the analysis (Fig. 8b–e). These analyses were highly supportive of our neural lineage predictions and demonstrated that the clustering groups identified in silico are robust and lead to novel lineage discovery as well as generate testable hypotheses to take back to the worm in vivo (Fig. 8f).

## Discussion

### In silico analysis as a new approach for elucidating planarian ASC lineages

Here we demonstrate the usefulness of computational techniques for predicting lineages from single cell transcriptomes in planarians. Waterfall was applied to hierarchically clustered single-cell transcriptome data to identify a neural stem cell population, the νNeoblasts, within the X1 FACS gate and predicted a neural lineage trajectory in planarian heads. Subsequent in situ hybridization experiments revealed neural expression patterns for several genes enriched in νNeoblasts and pseudotime analysis predicted that the expression of many of these ν-genes increases as differentiation progresses along the neural lineage. The in silico analyses also predicted the presence of a novel population of *piwi-2*^+^*piwi-1*^lo^ stem cells in the head, which was subsequently validated by dFISH. Previously, planarian stem cell studies have focused almost exclusively on *piwi-1*^+^ cells; thus, this finding should be considered for future studies, as exclusion of *piwi-1*^-^ cells may result in an incomplete view of the planarian stem cell compartment and biased interpretation of experimental results.

Although the focus here was on neural lineage progression, the Waterfall pipeline has led to the identification of a novel X2 lineage in the planarian head that was not enriched with neural gene sets. This illustrates the usefulness of pseudotime analysis for predicting lineages from highly heterogeneous scRNAseq datasets and for identifying new candidate genes for lineage specification, even without first selecting for a specific lineage by transgenic labeling. In future studies, it will be interesting to apply the Waterfall analysis pipeline to scRNAseq data acquired from whole-body samples and during brain regeneration to gain insights into the molecular timing of lineage specification in an injury context. Upon unbiased scRNAseq of thousands of cells in the future, we predict that every cell lineage in planarians can be dissected by computational means.

### Stem cell hierarchies in planarians and detection of neoblast classes

The concept of stem cell hierarchies has not been assessed to great depth in planarians due to the lack of transgenic lineage tracing. Here pseudotime analysis has predicted a neural lineage that progresses sequentially through three major X1 subgroups (Group 2, Group 1, and νNeoblasts)*.* In a recent study on planarian stem cell heterogeneity, σNeoblasts have been proposed to give rise to the ζNeoblasts of the epithelial lineage and potentially γNeoblasts of the gut lineage, speaking to their pluripotent nature and leaving open the possibility that other lineages may also extend from this stem cell class [20]. The expression of different tissue-specific markers in Group 1 may suggest that it is primed for differentiation along multiple lineages, with the enrichment of neural gene sets a consequence of collecting only cells from the head region. As displayed in Figs. 2d and 3b, Group 1 cells expressed the highest levels of the σNeoblast marker *soxP-2* and also expressed other genes previously shown by [20] to be enriched in σNeoblasts, raising the possibility that Group 1 cells are members of the σ-class. However, analysis of additional planarian scRNAseq data published by [40] questions the specificity of these previously published σNeoblast markers. As demonstrated in Additional file 5: Figure S4, the previously identified σNeoblast markers are in fact expressed evenly across all three neoblast classes identified by [40], unlike the ζ marker *zfp-1* and γ marker *hnf4*, which are largely specific to their respective classes. Thus, are σNeoblasts a truly distinct neoblast class or simply a collection of non-ζ and non-γ cells? Further, the proposition that σNeoblasts give rise to ζNeoblasts is based on the ability of X1 cells obtained from *zfp-1*(RNAi) animals to reconstitute the ζ-class when grafted into irradiated hosts with no stem cells [20]. Unfortunately, due to the technical limitations in isolating specific cell types, it is impossible to know precisely which types of neoblasts (σ, γ, ν, or other currently unidentified classes) gave rise to the newly formed ζNeoblasts. This is not to suggest that previous conclusions were unfounded but rather to highlight the limitations of current techniques for lineage analysis and the need for a new, unbiased approach for studying lineages in planarians. In addition, another σNeoblast marker, *znf-91*, identified by Wurtzel et al. [40] was found to be one of the top νNeoblast-enriched transcription factors and is primarily expressed in the brain and ventral nerve cords (Fig. 6), suggesting that some σNeoblasts may be misclassified νNeoblasts. Thus, due to the lack of specific markers, it is difficult to conclude whether previously described neoblast classes are represented in our dataset. Nevertheless, a connection between Group 1 and σNeoblasts can be drawn based on the predicted pluripotency of these two X1 groups, as presented by the PCA plots and model of proposed lineages in Fig. 8. The fact that different lineages appear to originate from Group 1/σNeoblasts supports this connection and supports the relationship between σNeoblasts and the ζNeoblasts/epithelial lineage despite the uncertainty mentioned above. That being said, the presence of additional heterogeneity within Group 1/σNeoblasts cannot be ruled out and may be resolved in future scRNAseq studies by using cells isolated from different regions of the planarian or during regeneration.

In addition to the neoblast classes discussed above, a relatively rare cell type, the clonogenic neoblasts (cNeoblasts), has been demonstrated to have the self-renewal capacity to re-populate the entire stem cell compartment following irradiation and can give rise to cells of all tissues [17, 35]. Such a stem cell would be expected to reside upstream of the Group 1/σNeoblasts on the stem cell hierarchy, leading to the hypothesis that Group 2 cells may be cNeoblasts (Fig. 8f). Interestingly, fewer transcripts are typically detected in Group 2 cells compared with Group 1 cells (Additional file 9: Figure S7). While highly speculative, this speaks to the idea that Group 2 may represent a less active, or possibly quiescent, stem cell population under homeostatic conditions. Although some cell cycle markers were detected in some Group 2 cells, it is not unreasonable to hypothesize that the massive injury caused by decapitating the worms prior to FACS resulted in the activation of this stem cell population in the short time period from amputation to sorting (30–60 min). It will be interesting to test these hypotheses in future studies by RNAi knockdown of group-specific candidate genes to better understand the nature of these stem cell subgroups and how they behave in both homeostatic and regenerative contexts in planarians.

### No prediction of the epithelial lineage in the head

From the results presented here, it is clear that Waterfall can be used as an efficient way to determine novel lineage trajectories, leading to testable hypotheses. However, from our regionalized X1 and X2 cells from the head, it did not predict the epithelial lineage that is already known to exist in planarians. For example, it has been shown that *zfp-1*^+^ ζNeoblasts give rise to *prog-1/2*^+^ and *agat-1*^+^ epithelial progenitors [20]. This lineage physically exists in the head by WISH, yet Waterfall did not pull the lineage out of our scRNAseq dataset. There are three possible explanations for this: (1) Waterfall was not sensitive enough to detect this lineage; (2) the low percentage of cells that express these progenitor markers was too small in the context of 168 cells used in this study; or (3) the X1 stem cells that give rise to epithelial progenitors are not prevalent in the head. First, the abundance of *prog-1/2*^+^ epithelial progenitors in the X2 gate is very low (only 8.5 % of X2s are early epithelial progenitors [47]) and the number detected in this study was 5/72 X2s, or 7 %. This is a very low number in the context of our total cells and neither *prog-1* nor *prog-2* were identified in the top 1000 variably expressed genes. Furthermore, no study has been able to show that epithelial progenitors are actually born in the head and they may instead be born more posterior and migrate forward, similar to eye progenitors [45]. Second, as illustrated in Additional file 6: Figure S5, when we incorporated 245 additional cells sequenced in [40] from the body of the animal and used the top 1000 variable transcripts from this combined dataset, our analysis pipeline readily predicts the known order of epithelial lineage differentiation described by previous works [18, 20, 47]. This proof of principle example provides confidence in the ability of this technique and analysis pipeline to reconstruct planarian neoblast lineages. Thus, we propose that the epithelial lineage was not predicted in our dataset due to a combination of sequencing cells only from the head region and the total number of cells sequenced.

## Conclusions

The large number and accessibility of ASCs and ASC progeny in planarians, coupled with their incredible capacity for regeneration, has branded this animal as a key model system for stem cell and regeneration biology. Without transgenics, however, elucidating the mechanisms of tissue turnover and regeneration in vivo has been challenging. Here we show for the first time that, with recent advances in single cell technology and bioinformatics modeling, it is possible to discover ASC lineages in planarians de novo via pseudotime analysis of single cell transcriptomes. This approach has identified a new neural stem cell population, the νNeoblasts, and has predicted the existence of a novel X2 lineage in planarian heads (Fig. 8f). Overall, this study demonstrates the usefulness of in silico lineage tracing with Waterfall for studying the progressive differentiation of planarian adult stem cells along multiple lineages. This approach can be applied to regeneration studies in planarians in order to gain insights into the mechanisms regulating ASC fate decisions.

## Methods

### Single-cell FACS and cDNA library preparation

FACS was performed as previously described [18]. Single-cell cDNA libraries were prepared using the Smartseq2 protocol, as previously described [26, 48]. See Additional file 10: Supplemental data file 1 for a detailed protocol.

### Sequencing and read alignment

Single-cell libraries were sequenced to an average depth of 4.5 million single end 50-bp reads on an Illumina HiSeq2500 with v4 chemistry and the data have been uploaded under NCBI Gene Expression Omnibus (GEO) project GSE79866. Reads were aligned to the *S. mediterranea* SmedASXL transcriptome assembly under NCBI BioProject PRJNA215411 using bowtie2 [27] with 15-bp 3′ trimming. Raw read counts (Additional file 11: Supplemental data file 5) were imported into R (version 3.2.2) [49] as a matrix with transcripts as rows and cells as columns and normalized with DESeq [50]. See Additional file 11: Supplemental data file 5 for raw single cell counts.

### Selection of the top 1000 highly variable transcripts

The normalized counts data were Winsorized to prevent counts from the two most extreme individual cells from contributing to gene selection. Row means and coefficients of variation (CV) were calculated and log-transformed, then plotted as a smooth scatterplot using the smoothScatter function from the graphics package in R. A regression line with 95% confidence intervals was fit to the scatterplot using the statmod package [51] and transcripts were ranked by the significance of their deviation from the fit. See Additional file 3: Supplemental data file 3 for a ranked list of the top 1000 highly variable transcripts. A new counts matrix was created (hvg1000.RData), which included the top 1000 transcripts from the ranked list and their normalized read counts in each cell. These counts data were used for all subsequent cluster analyses. The full dataset was also saved as a RData file (full_dataset.RData) for subsequent use in the heatmap and Waterfall pipeline. See Additional file 12: Supplemental data file 2 for a vignette and Additional file 13.

### Cluster analysis

A Euclidean distance matrix was computed for the hvg1000 data matrix using the dist function from the stats package in R with default parameters. Hierarchical clustering was then performed using the hclust function with the parameter method = “ward.D2” and the results were plotted using the plot function. The cutree function was used to cut the dendrogram into k = 10 groups, which allowed for separation into several distinct X1 and X2 subgroups. The rect.hclust function was used to add colored boxes around the subgroups on the hclust dendrogram. t-SNE was performed using the R implementation (Rtsne) [30]. The Rtsne function was applied to the hvg1000 counts matrix with default parameters. The results were plotted with the color of the data points corresponding to the group colors from the hclust dendrogram or by FACS gate. The heatmaps were produced using the heatmap.3 code available from https://raw.githubusercontent.com/obigriffith/biostar-tutorials/master/Heatmaps/heatmap.3.R with minor modifications (provided as Additional file 14: Supplemental data file 7). See Additional file 15: Supplemental data file 6 for the counts used in Fig. 2d.

### Gene set enrichment analysis

GSEA was performed as previously described using planarian genes with a reciprocal top BLAST hit in the top five hits to mouse homologs when e < 1e^-4^ and freely available GSEA software [32] (http://www.broadinstitute.org/gsea/). The bulk X1 data were obtained from NCBI GEO (accession numbers GSE68581 and GSE37910).

### Waterfall analysis

Waterfall analysis was performed by following the vignette and using the source code available from Shin et al. [22]. PCA was initially performed on the hvg1000 dataset including all hierarchical clustering groups and the mst.of.classification Waterfall function was used to plot a MST trajectory. Single lineage trajectories were identified as described in the main text. Prior knowledge of the planarian stem cell compartment and CNS was used to determine the direction of the MST trajectory; this was visualized by plotting the PCA results and computing the size of the data points using the scale_row.foo Waterfall function. The pseudotimeprog.foo function was then called to calculate a pseudotime value for each cell. The y-axis in pseudotime plots represents the distance of each cell to its nearest k-means center. The pseudotime.foo function was used to plot the expression levels of specific genes over pseudotime. See Additional file 12: Supplementary data file 2 for a vignette. Analyses including data from [40] included only single cells isolated at 0 h post-injury and these cells were grouped by their cluster assignment in [40] (accession number SRA:PRJNA276084).

### Animal husbandry

Asexual individuals of *S. mediterranea* CIW4 strain were reared as previously described [52].

### Cloning

Transcripts enriched in νNeoblasts were identified by performing differential expression analysis using the SCDE R package [28] and cloned using forward and reverse primers into T4P vectors as previously described [53] and these vectors were subsequently used as PCR templates for the production of riboprobes as previously described [54]. Previously undescribed planarian transcripts were named by their top reciprocal blast hit to mouse. The transcripts cloned in this manuscript are available in Additional file 8: Supplementary data file 4.

### BrdU, in situ hybridization, and image acquisition

BrdU (Sigma B5002-5G, 25 mg/ml) was dissolved in 50 % ethanol and injected into the gut of animals. Animals were fixed 4 h later and BrdU was stained as previously described [20]. In situ hybridizations were performed as previously described [18, 55]. Colorimetric WISH samples were imaged on a Leica M165 fluorescent dissecting microscope. dFISH and tFISH samples were imaged on a Leica DMIRE2 inverted fluorescence microscope with a Hamamatsu Back-Thinned EM-CCD camera and spinning disc confocal scan head with Volocity software. Raw images were opened in ImageJ and saved as tiffs and resolution, brightness, and contrast were adjusted in Adobe Photoshop.

### Availability of supporting data

The scRNAseq data set supporting the results of this article were uploaded to NCBI GEO, accession number GSE79866. The whole-worm bulk X1 data sets are available from NCBI GEO, accession numbers GSE68581 (http://www.ncbi.nlm.nih.gov/geo/query/acc.cgi?acc=GSE68581) and GSE37910 (http://www.ncbi.nlm.nih.gov/geo/query/acc.cgi?acc=GSE37910). The *S. mediterranea* SmedASXL transcriptome assembly is available from NCBI BioProject PRJNA215411 (http://www.ncbi.nlm.nih.gov/bioproject/?term=PRJNA215411).

## Additional files

Additional file 1: Figure S1. FACS gates used for isolating single cells. **a** Unirradiated and **b** irradiated FACS plots. X1 and X2 gates were set based on Hoechst red vs. blue fluorescence detection as previously described [18, 32]; 20,000 cells are plotted. (PNG 422 kb) Additional file 2: Figure S2. Markers of known tissue lineages are detected among the bulk and scRNAseq samples. Heatmaps displaying log2 normalized counts of known tissue-specific markers in **a** bulk X1 and X2 samples (200 cells each) and **b** select single cells (in columns). (PNG 169 kb) Additional file 3: A ranked list of the top 1000 highly variable transcripts with means, coefficients of variation, and the hierarchical clustering group in which the average expression was highest. (XLSX 56 kb) Additional file 4: Figure S3. PCA plot including all ten hierarchical clustering groups. This method did not clearly distinguish individual clusters of cell types. (PNG 107 kb) Additional file 5: Figure S4. Previously described σNeoblast markers are not specific to the σ-class. Plots were made using an online single cell RNAseq resource published by [40] (http://radiant.wi.mit.edu/app/). Legend of neoblast classes in top right. (PNG 245 kb) Additional file 6: Figure S5. Pseudotime analysis correctly reconstructs the epithelial lineage. **a** PCA plot including the ζNeoblast, early epithelial, and late epithelial groups identified in [40]. *Red line*, MST connecting k-means centers; *grey arrow*, direction of lineage progression. **b** Pseudotime plot for the epithelial lineage. *Red line*, linearized MST connecting k-means centers; *grey arrow*, direction of lineage progression; *y-axis*, distance of cell to its nearest k-means center. **c** Summary model of the epithelial lineage. **d–f** Expression levels of previously described ζNeoblast (**d**), early epithelial progeny (**e**), and late epithelial progeny (**f**) markers. *Red line*, local polynomial regression fit; *shaded region*, 95 % confidence interval. (PNG 539 kb) Additional file 7: Figure S6. Additional ν-enriched genes. Colorimetric WISH for seven additional transcripts enriched in the νNeoblasts showed strong brain expression. Dorsal view, anterior up, scale bars = 100 μm. (PNG 760 kb) Additional file 8: A table listing the newly described genes and top BLASTx hits. (XLSX 33 kb) Additional file 9: Figure S7. The number of transcripts detected decreases with increasing differentiation state. **a** The number of transcripts detected versus read depth for each single cell sample shows no correlation. Pearson correlation = 0.1869, *R* ^2^ = 0.03494. **b, c** Number of transcripts detected (*left*) or number of reads (*right*) plotted along pseudotime for the X2 (**b**) and neural (**c**) lineages. The *red dashed line* indicates the start of lineage commitment. *Solid red line*, local polynomial regression fit; *shaded region*, 95 % confidence interval. (PNG 297 kb) Additional file 10: A detailed protocol for single cell FACS and RNA-seq. (DOCX 39 kb) Additional file 11: A table of raw read counts obtained for all 168 single cell samples aligned to the SmedASXL planarian transcriptome assembly. (TXT 23858 kb) Additional file 12: A vignette for reproducing the computational analyses. (TXT 11 kb) Additional file 13: An annotation file for use with the vignette. (TAB 999 bytes) Additional file 14: The modified heatmap source code. (R 16 kb) Additional file 15: A table of normalized counts for the genes used in Fig. 2d for cells in Group 1, Group 2, and νNeoblasts. (TXT 13 kb)

## Abbreviations

ASC: adult stem cell
BrdU: bromodeoxyuridine
CNS: central nervous system
dFISH: double fluorescent in situ hybridization
FACS: fluorescence-activated cell sorting
FDR: false discovery rate
FISH: fluorescent in situ hybridization
GEO: Gene Expression Omnibus
GSEA: gene set enrichment analysis
MST: minimum spanning tree
PCA: principle components analysis
RNAi: RNA interference
scRNAseq: single-cell RNA-deep sequencing
tFISH: triple fluorescent in situ hybridization
t-SNE: t-Distributed Stochastic Neighbor Embedding
WISH: whole-mount in situ hybridization
