# Supplementary figures and images for: In silico lineage tracing through single cell transcriptomics identifies a neural stem cell population in planarians
Source: Genome Biol. 2016 Apr 27;17:87. doi: 10.1186/s13059-016-0937-9 (PMC4858873; doi:10.1186/s13059-016-0937-9)

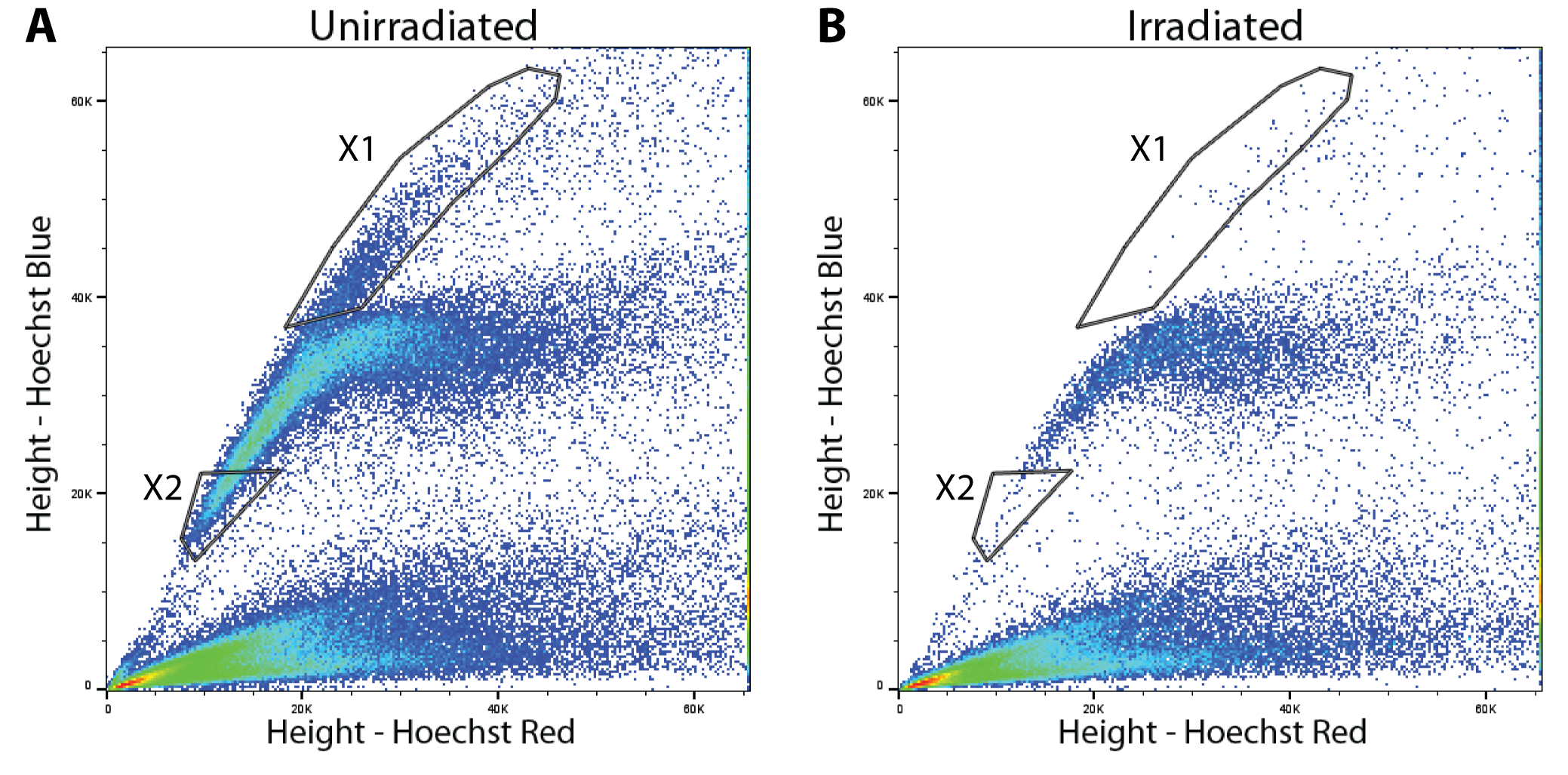

Supplement: Additional file 1: Figure S1. — FACS gates used for isolating single cells. a Unirradiated and b irradiated FACS plots. X1 and X2 gates were set based on Hoechst red vs. blue fluorescence detection as previously described [18, 32]; 20,000 cells are plotted. (PNG 422 kb) [file 13059_2016_937_MOESM1_ESM.png]

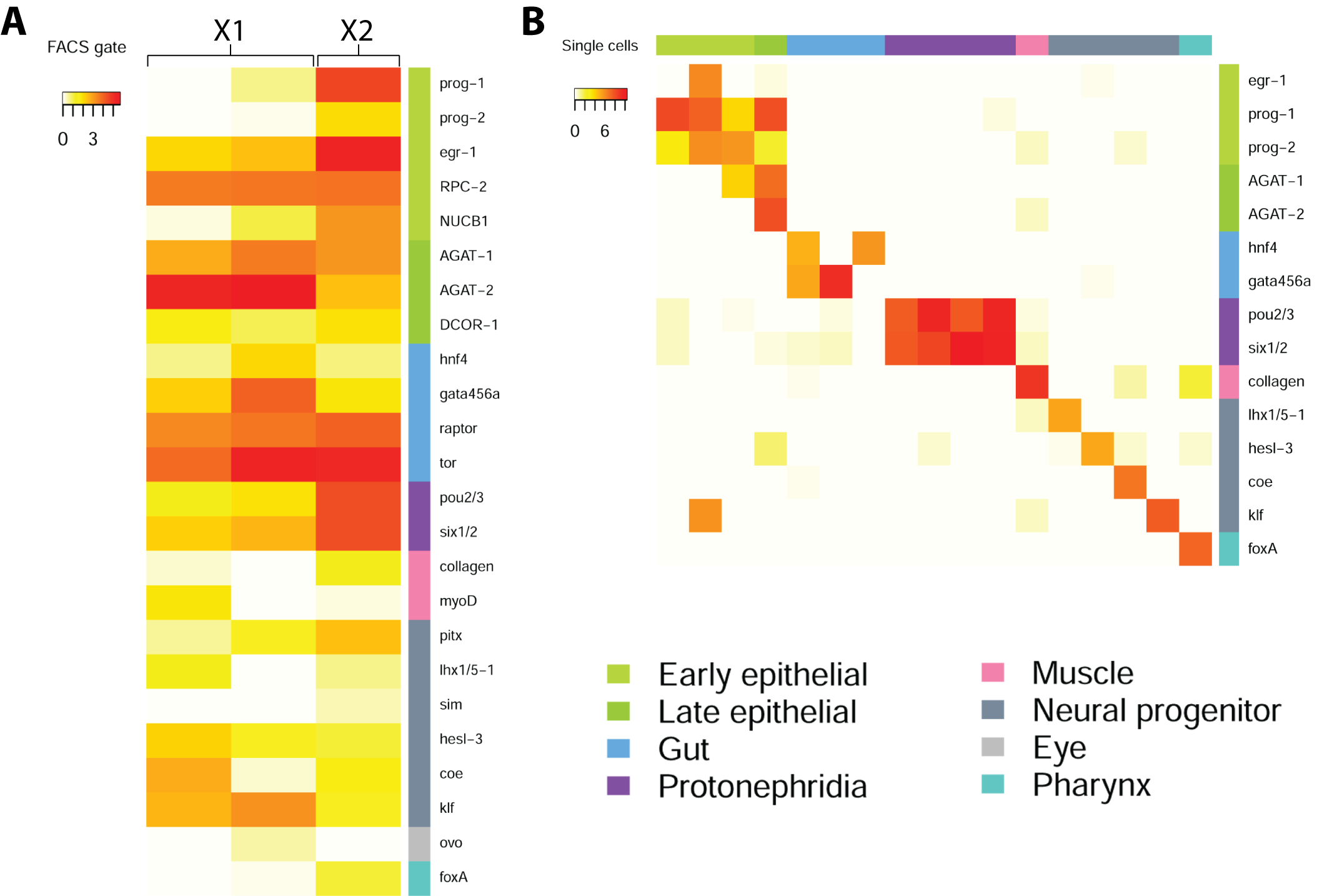

Supplement: Additional file 2: Figure S2. — Markers of known tissue lineages are detected among the bulk and scRNAseq samples. Heatmaps displaying log2 normalized counts of known tissue-specific markers in a bulk X1 and X2 samples (200 cells each) and b select single cells (in columns). (PNG 169 kb) [file 13059_2016_937_MOESM2_ESM.png]

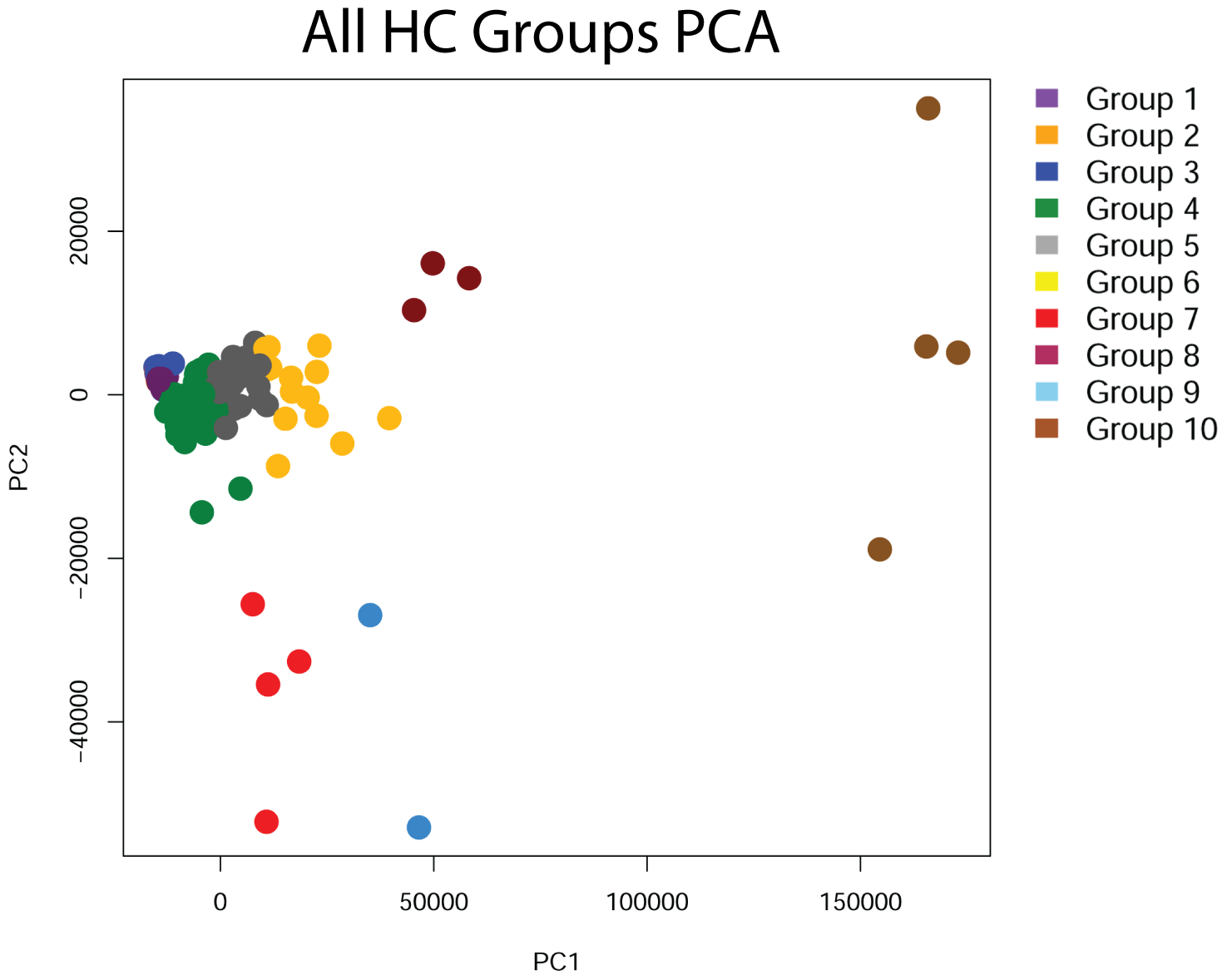

Supplement: Additional file 4: Figure S3. — PCA plot including all ten hierarchical clustering groups. This method did not clearly distinguish individual clusters of cell types. (PNG 107 kb) [file 13059_2016_937_MOESM4_ESM.png]

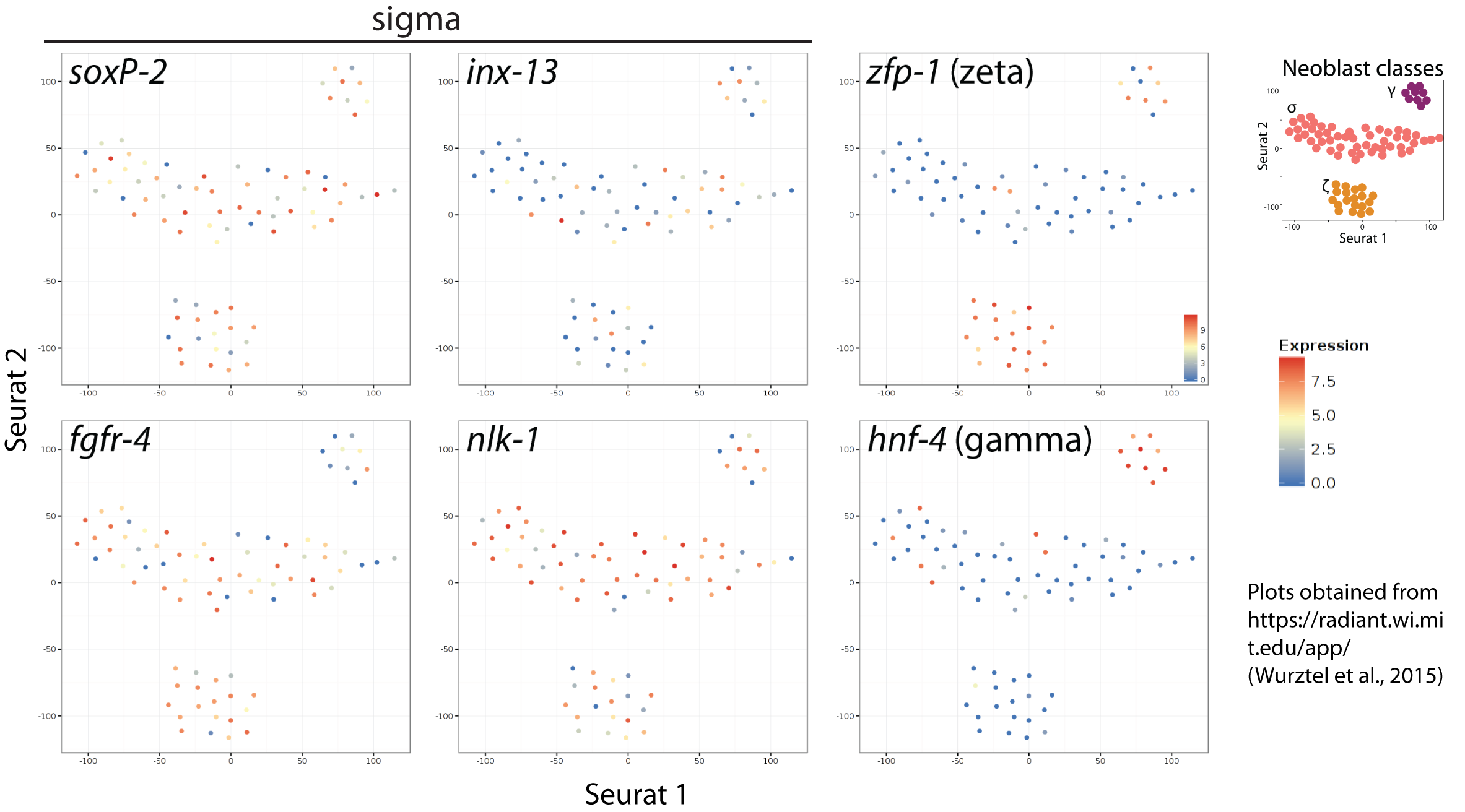

Supplement: Additional file 5: Figure S4. — Previously described σNeoblast markers are not specific to the σ-class. Plots were made using an online single cell RNAseq resource published by [40] (http://radiant.wi.mit.edu/app/). Legend of neoblast classes in top right. (PNG 245 kb) [file 13059_2016_937_MOESM5_ESM.png]

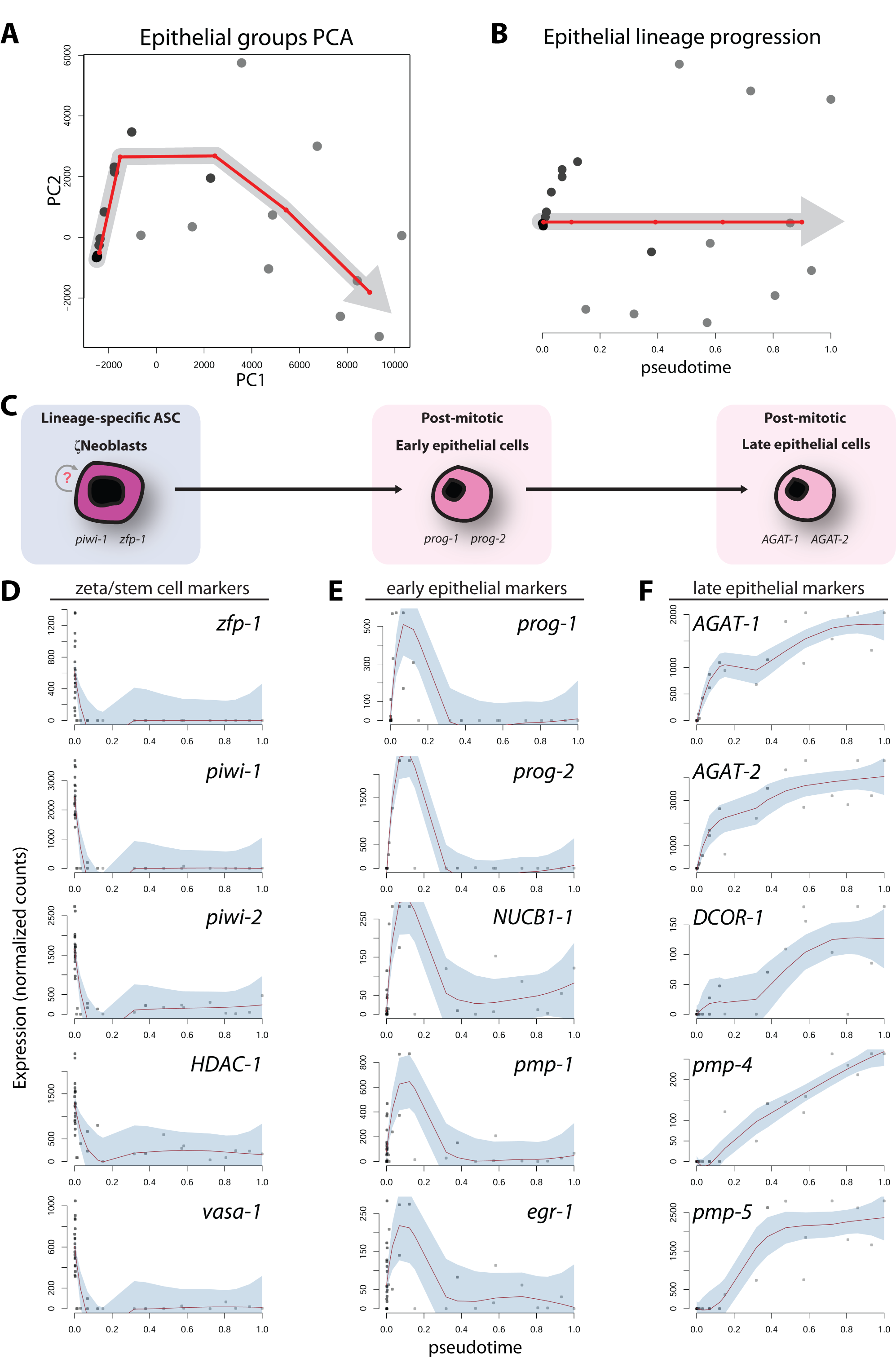

Supplement: Additional file 6: Figure S5. — Pseudotime analysis correctly reconstructs the epithelial lineage. a PCA plot including the ζNeoblast, early epithelial, and late epithelial groups identified in [40]. Red line, MST connecting k-means centers; grey arrow, direction of lineage progression. b Pseudotime plot for the epithelial lineage. Red line, linearized MST connecting k-means centers; grey arrow, direction of lineage progression; y-axis, distance of cell to its nearest k-means center. c Summary model of the epithelial lineage. d–f Expression levels of previously described ζNeoblast (d), early epithelial progeny (e), and late epithelial progeny (f) markers. Red line, local polynomial regression fit; shaded region, 95 % confidence interval. (PNG 539 kb) [file 13059_2016_937_MOESM6_ESM.png]

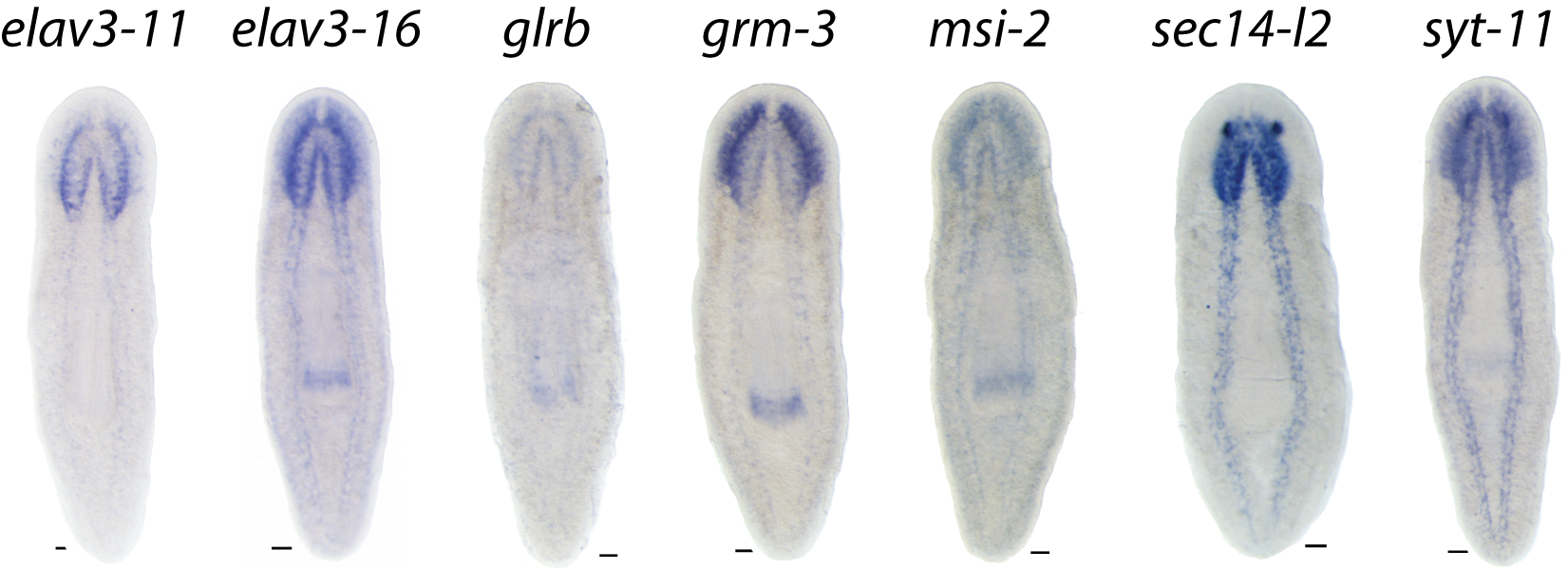

Supplement: Additional file 7: Figure S6. — Additional ν-enriched genes. Colorimetric WISH for seven additional transcripts enriched in the νNeoblasts showed strong brain expression. Dorsal view, anterior up, scale bars = 100 μm. (PNG 760 kb) [file 13059_2016_937_MOESM7_ESM.png]

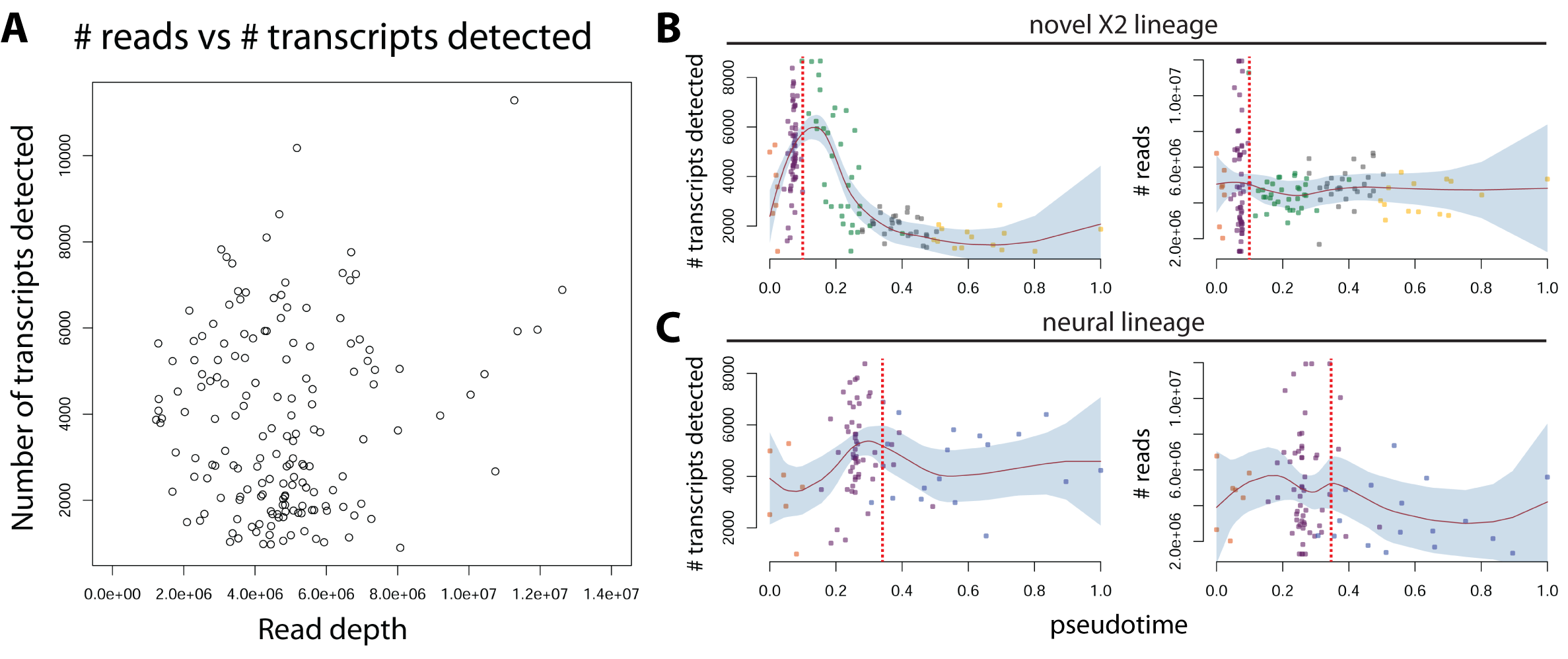

Supplement: Additional file 9: Figure S7. — The number of transcripts detected decreases with increasing differentiation state. a The number of transcripts detected versus read depth for each single cell sample shows no correlation. Pearson correlation = 0.1869, R 2 = 0.03494. b, c Number of transcripts detected (left) or number of reads (right) plotted along pseudotime for the X2 (b) and neural (c) lineages. The red dashed line indicates the start of lineage commitment. Solid red line, local polynomial regression fit; shaded region, 95 % confidence interval. (PNG 297 kb) [file 13059_2016_937_MOESM9_ESM.png]
