# Supplementary material for: In silico lineage tracing through single cell transcriptomics identifies a neural stem cell population in planarians
Source: Genome Biol. 2016 Apr 27;17:87. doi: 10.1186/s13059-016-0937-9 (PMC4858873; doi:10.1186/s13059-016-0937-9)
Supplement: Additional file 10: — A detailed protocol for single cell FACS and RNA-seq. (DOCX 39 kb) [file 13059_2016_937_MOESM10_ESM.docx]

**Single cell RNA-seq of planarian head X1 and X2 cells by FACS sorting and SmartSeq2**

**Reagents:**

FACS

- CMF (Reddien et al., Science, 2005 and http://pearsonlab.ca/protocols/Planarian_FACSSORTING.pdf)
- Hoechst 342 (Thermo Scientific 62249)
- PI (Molecular Probes P3566)

SmartSeq2

- PBS, no Ca^2+^, no Mg^2+^ (Roche 11666789001)
- Triton X-100 (Biobasic TB0198)
- dNTP mix, 10mM each (Thermo Scientific R0191)
- 5X First-strand buffer (Invitrogen 18090010)
- DTT (Invitrogen 18090010)
- Superscript IV reverse transcriptase (Invitrogen 18090010)
- RNAseOUT (Invitrogen 10777-019)
- Betaine (Sigma-Aldrich, cat. no. 61962)
- MgCl_2_ (Sigma-Aldrich, cat. no. M8266)
- Nuclease-free water (Ambion AM9932)
- KAPA HiFi HotStart ReadyMix 2X (KAPA Biosystems KK2601)
- Agencourt Ampure XP beads (Beckman Coulter A63881)
- EB solution, 10mM Tris-Cl pH8.5 (Qiagen 19086)
- Nextera XT DNA sample prep kit, 96 samples (Illumina FC-131-1096)
- Nextera XT 24-index kit, 96 samples (Illumina FC-131-1001)
- Template-switching oligo with LNA

5’-AAGCAGTGGTATCAACGCAGAGTACATrGrG+G-3’ (Exiqon)

- Oligo-dT(20)VN – 5’-AAGCAGTGGTATCAACGCAGAGTACT_20_VN-3’ (IDT)
- ISPCR oligo – 5’-AAGCAGTGGTATCAACGCAGAGT-3’ (IDT)

**Single cell RNA-seq of planarian head X1 and X2 cells by FACS sorting and SMART-seq2**

**Procedure**

**Preparing single-cell samples by FACS TIMING 2 h**

1. Collect 30-60 planarian heads into a 1.5ml eppendorf tube.
2. Wash 2x with cold CMF.
3. Gently dounce worms with a sterile plastic pestle until big fragments are all broken down.
4. Assemble the filter setup with a 20um filter. Pre-wet the filter with CMF.
5. Pour the cells through the filter into a new 50ml conical tube. Cells may need a little coaxing to get through.
6. Add Hoechst 342 (use 25 ug/ml of cells) and invert immediately to avoid precipitate. Incubate for 20 min in the dark.
7. While cells are incubating:

- Dilute the oligo-dT primer to 10 uM with nuclease-free water

Then prepare the lysis buffer + oligo-dT primer + dNTP mix (= **Lysis Mix**) by combining (for 96 samples + extra):

- 10 ul RNaseOUT
- 190 ul 0.2% (vol/vol) Triton X-100
- 100 ul oligo-dT primer (10 uM)
- 100 ul dNTP mix

Transfer 4 ul of Lysis Mix into each tube of a 96-tube plate.

1. Spin down cells (300g for 5 min, low brake). Resuspend cells in 1-2 ml CMF.
2. Add PI (use 4 ug/ml of cells) and place on ice.
3. Sort X1 and X2 cells directly into 96-tube plates containing Lysis Mix. Plates can be stored at -80°C.

**Single-cell lysis TIMING 5 min**

**NOTE:** 8 cDNA libraries were prepared at a time.

1. Break off an 8-tube strip from the 96-tube plate. Freeze the remainder of the plate at -80°C.
2. Spin down the samples to ensure all contents are collected at the bottom of each tube (700g for 10 sec at RT). Immediately place on ice.
3. Incubate the samples at 72°C for 3 min and immediately place back on ice.
4. Spin down the samples (700g for 10 sec at RT). Immediately place back on ice.

**Reverse Transcription TIMING 3 h**

1. Prepare the **RT mix** as follows (for 9 reactions):

**CRITICAL STEP** Thaw all reagents in advance and assemble the RT mix while performing denaturation (Step 12) to minimize bias.

| Component | Volume (ul) | Final Concentration |
| --- | --- | --- |
| SuperScript IV reverse transcriptase (200 U/ul) | 4.5 | 100 U |
| RNAseOUT (40 U/ul) | 2.25 | 10 U |
| First strand buffer (5x) | 18 | 1x |
| DTT (100 mM) | 4.5 | 5 mM |
| Betaine (5 M) | 18 | 1 M |
| MgCl_2_ (1 M) | 0.54 | 6 mM |
| TSO (100 uM) | 0.9 | 1 uM |
| Nuclease-free water | 5.31 | --- |
| Total volume per tube | **6.0** | **---** |

1. Add 6 ul of the RT mix to each sample to obtain a final reaction volume of 10 ul. Mix the reaction gently by pipetting up and down a few times without forming bubbles.
2. Spin down the samples (700g for 10 sec at RT) to collect liquid and incubate the reaction in a thermal cycler with a heated lid to carry out the **First-strand reaction**, as below:

**CRITICAL STEP** Use a thermal cycler with a heated lid set to 105°C for all incubations throughout this protocol.

| Cycle | Temperature (°C) | Time | Purpose |
| --- | --- | --- | --- |
| 1 | 50 | 90 min | RT and template-switching |
| 2-11 | 55 | 2 min | Unfolding of RNA secondary structures |
|  | 50 | 2 min | Completion/continuation of RT and template-switching |
| 12 | 70 | 15 min | Enzyme inactivation |
| 13 | 4 | Hold | Safe storage |

**PCR preamplification TIMING 3 h**

1. Prepare the **PCR mix** as follows (for 9 reactions):

| Component | Volume (ul) | Final Concentration |
| --- | --- | --- |
| KAPA HiFi HotStart ReadyMix (2x) | 112.5 | 1x |
| ISPCR primers (10 uM) | 2.25 | 0.1 uM |
| Nuclease-free water | 20.25 | --- |
| Total volume per tube | **15** | **---** |

1. Add 15 ul of the PCR mix to each sample (i.e. the First-strand reaction) to obtain a final reaction volume of 25 ul. Mix by vortexing, then spin down (700g for 10 sec at RT).
2. Perform the PCR in a thermal cycler, as below:

| Cycle | Denature | Anneal | Extend | Hold |
| --- | --- | --- | --- | --- |
| 1 | 98**°**C, 10 s | --- | --- | --- |
| 2-19 | 98**°**C, 20 s | 67**°**C, 15 s | 72**°**C, 6 min | --- |
| 20 | --- | --- | 72**°**C, 5 min | --- |
| 21 | --- | --- | --- | 4**°**C |

**CRITICAL STEP** The number of PCR cycles depends on the input amount of RNA. 18 cycles is typically used for eukaryotic cells to obtain ~1-30 ng of amplified cDNA. The number of cycles can be increased for smaller cells (with less RNA content).

**PAUSE POINT** PCR product can be stored at -20**°**C or -80**°**C for 6 months or longer

**PCR purification TIMING 45 min**

1. Before starting purification, equilibrate the Ampure XP beads at RT for 15 min then vortex for several seconds.
2. Add 25 ul of Ampure XP beads (1:1 ratio) to each sample and mix by pipetting up and down until the solution appears homogeneous. Incubate for 8 min at RT to let the DNA bind to the beads.

**CRITICAL STEP** Do not increase the volume of beads above the 1:1 ratio.

1. Place the 8-tube strip on the magnetic stand for 5 min or until the solution is clear and beads have been collected at one side of the tubes.
2. Carefully remove the liquid without disturbing the beads.
3. Wash the beads 200 ul of 80% (vol/vol) ethanol. Incubate the samples for 30 sec and then remove the ethanol. Repeat once more.

**CRITICAL STEP** Freshly prepare the ethanol solution every time.

1. Remove any trace of ethanol and let the beads dry completely by leaving the strip at RT for 2 min.

**CRITICAL STEP** Avoid overdrying the beads as this will make resuspension more difficult.

1. Add 17.5 ul of EB solution (or nuclease-free water). Mix ten times to resuspend the beads.
2. Incubate the strip **off** the magnetic stand for 2 min.
3. Incubate the strip **on** the magnetic stand for 2 min or until the solution appears clear and beads have been collected to one side of the tubes.
4. Set the volume of the pipette to 15 ul. Collect the supernatant without disturbing the beads and transfer to a fresh 8-tube strip.

**CRITICAL STEP** Avoid aspirating the whole volume. Leaving 2.5 ul behind ensures that bead carryover is kept to a minimum.

**Quantifying dsDNA by Qubit TIMING 30 min**

1. Set up ten 0.5 ml Qubit assay tubes (8 for samples plus 2 standards). Label the tube lids

**CRITICAL STEP** Do not label the sides of the tubes as this will interfere with the sample read.

1. Prepare the Qubit working solution by diluting the Qubit dsDNA HS Reagents 1:200 in Qubit dsDNA HS Buffer. Each standard tube requires 190 ul of Qubit working solution and each sample tube requires 180-199 ul.

**CRITICAL STEP** We want to use a minimal amount of cDNA for this quantification, i.e. 1 ul. For 8 samples plus 2 standards, dilute 10 ul of Qubit reagent in 1990 ul of Qubit buffer.

1. Add 190 ul of Qubit working solution to each standard tube, then add 10 ul of each Qubit standard into one of the tubes. Mix by vortexing briefly. Do not create bubbles.
2. Add 199 ul Qubit working solution to each sample tube, then add 1 ul of each sample to the appropriate tube. Mix by vortexing briefly.
3. Allow all tubes to incubate at RT for 2 min.
4. Read samples on a Qubit 2.0 fluorometer as follows:

- On the Home screen of the Qubit 2.0 fluorometer, press **DNA**, then select **dsDNA High Sensitivity** as the assay type. The Standards screen is displayed.
- On the Standards screen, press **Yes** to read the standards.
- Insert the tube containing Standard #1 into the sample chamber, close the lid, then press **Read**. When the reading is complete (~3 sec), remove Standard #1.
- Insert the tube containing Standard #2 into the sample chamber, close the lid, then press **Read**. When the reading is complete, remove Standard #2.
- When the calibration is complete, the instrument displays the Sample screen.
- Insert a sample tube into the sample chamber, close the lid, the press **Read**. When the reading is complete (~3 sec), remove the sample tube.
  - The instrument displays the results on the Sample screen. The value displayed corresponds to the concentration of the diluted sample in ng/ml. Record this value.
  - Repeat for all samples.

1. Calculate the sample concentration (ng/ml) as follows:

- Concentration of sample = QF value x (200/n)
  - Where QF value = the value given by the Qubit 2.0 fluorometer and n = the number of ul of sample added to the assay tube

1. Calculate the volume of each sample corresponding to 1 ng of DNA.

**PAUSE POINT** PCR product can be stored at -20**°**C or -80**°**C.

**Tagmentation reaction (Nextera) TIMING 10 min**

1. Prepare reagents:

- Thaw ATM and TD on ice.
- Visually inspect NT to make sure there is no precipitate. Vortex if necessary.
- Gently mix all reagents by inverting the tubes 3-5 times, followed by a brief spin.
- Measure the concentration of DNA in each sample.

1. Label a new 8-tube strip “**NTA**” (Nextera XT Tagment Amplicon Plate).
2. Add 10 ul TD buffer to each tube.
3. Add 1 ng of input DNA to each tube of the NTA strip. Gently pipet up and down to mix. Top up to 15 ul total volume with nuclease-free water.
4. Add 5 ul ATM to each tube. Gently mix.
5. Seal the NTA plate with a strip caps. Centrifuge the NTA plate at 280g at 20°C for 1 min to collect reagents, if necessary.
6. Perform the tagmentation reaction in a thermal cycler, as below:

| Cycle | Temperature (°C) | Time |
| --- | --- | --- |
| 1 | 55 | 5 min |
| 2 | 10 | Hold |

**CRITICAL STEP** When the sample reaches 10°C proceed immediately to neutralization as the transposase is still active.

**Stripping Tn5 transposase off the tagmented DNA TIMING 5 min**

1. Carefully remove the caps and add 5 ul of NT buffer to each tube of the NTA strip. Pipet to mix.
2. Seal the NTA strip with strip caps and Incubate for 5 min at RT (spin if necessary: 280g at 20°C for 1 min). DNA is now ready for the final enrichment PCR.

**Amplification of adapter-ligated fragments TIMING 1 h**

1. Thaw the NPM and index primers (i5 and i7) on bench at RT (allow ~20 min). Then gently invert all tubes and spin briefly.

**CRITICAL STEP** Only thaw the index primers being used.

1. Add 15 ul NPM to each tube of the NTA strip.

**CRITICAL STEP** Since we are only processing 8 samples at a time, keep track of the index primer pairs used by recording on the Nextera XT Lab Tracking Form (available online).

1. Add 5 ul of index 2 primers (white caps) to each corresponding row of the NTA strip.
2. Using a multichannel pipet, add 5 ul of index 1 primers (orange caps) to each corresponding column of the NTA strip. Gently pipet up and down to mix.
3. Cover the NTA strip with strip caps and centrifuge at 280g at 20°C for 1 min.
4. Perform the PCR, as below:

| Cycle | Denature | Anneal | Extend | Hold |
| --- | --- | --- | --- | --- |
| 1 | --- | --- | 72**°**C, 3 min | --- |
| 2 | 95**°**C, 30 s | --- | --- | --- |
| 3-14 | 95**°**C, 10 s | 55**°**C, 30 s | 72**°**C, 30 s | --- |
| 15 | --- | --- | 72**°**C, 5 min | --- |
| 16 | --- | --- | --- | 4**°**C |

**PAUSE POINT** The NTA strip can remain on the thermal cycler overnight or can be stored at 4°C for up to 2 days.

**PCR Clean-up TIMING 45 min**

1. Bring the AMPure XP beads to RT and prepare fresh 80% ethanol.
2. Centrifuge the NTA strip at 280g at 20°C for 1 min to collect condensation.
3. Label a new 8-tube strip **CAA** (Clean Amplified Plate).
4. Using a multichannel pipet set to 50 ul, transfer the PCR product from the NTA strip to the CAA strip.
5. Vortex the AMPure XP beads for 30 seconds. Add 30 ul of beads to each tube in the CAA strip. Gently pipet to mix.
6. Incubate at RT **off** the magnetic stand for 5 min.
7. Incubate **on** the magnetic stand for 2 min or until the supernatant has cleared.
8. With the CAA strip on the magnetic stand, use a multichannel pipet to remove and discard the supernatant. Leave the CAA strip on the stand.
9. Wash the beads by adding 200 ul of 80% ethanol to each tube. Do not resuspend the beads. Incubate for 30 sec, then carefully remove the ethanol. Repeat.
10. Remove as much ethanol as possible without disturbing the beads, then allow beads to air-dry for 15 min.
11. Remove the CAA strip from the magnetic stand and add 52.5 ul of RSB to each tube. Gently mix and incubate at RT for 2 min.
12. Place the CAA strip on the magnetic stand for 2 minutes or until the supernatant has cleared.
13. Label a new 8-tube strip **CAN** (Clean Amplified NTA Plate).
14. Using a multichannel pipet, carefully transfer 50 ul of the supernatant from the CAA strip to the CAN strip.

**PAUSE POINT** The CAN strip can now be sealed with strip caps and stored at -20°C for up to 1 week.

**Final library quantification by Qubit TIMING 30 min**

1. Repeat steps 31-38.

**Sequencing**

1. Samples were submitted to the Donnelly Sequencing Center, Toronto, ON, CA and sequenced on an Illumina HiSeq2500 with v4 chemistry to a depth of 5 million 50 bp single-end reads.

**References**

Picelli S, Faridani O, Björklund Å, Winberg G, Sagasser S, Sandberg R: **Full-length RNA-seq from single cells using Smart-seq2**. *Nature Protocols* 2014, **9**:171–8110.1038/nprot.2014.006

Reddien PW, Oviedo NJJ, Jennings JR, Jenkin JC, Sánchez Alvarado A: **SMEDWI-2 is a PIWI-like protein that regulates planarian stem cells.** *Science*2005, **310**:1327–3010.1126/science.1116110

Pearson BJ, Sánchez Alvarado A: **A planarian p53 homolog regulates proliferation and self-renewal in adult stem cell lineages.** *Development*2010, **137**:213–2110.1242/dev.044297.
